# *Pax3/7* duplicated and diverged independently in amphioxus, the basal chordate lineage

**DOI:** 10.1038/s41598-018-27700-x

**Published:** 2018-06-20

**Authors:** Thomas B. Barton-Owen, David E. K. Ferrier, Ildikó M. L. Somorjai

**Affiliations:** 1 University of St Andrews, Gatty Marine Laboratory, Scottish Oceans Institute, East Sands, St Andrews, Fife, KY16 8LB UK; 2 University of St Andrews, Biomedical Sciences Research Complex, North Haugh, St Andrews, Fife, KY16 9ST UK

## Abstract

The *Pax3/7* transcription factor family is integral to developmental gene networks contributing to important innovations in vertebrate evolution, including the neural crest. The basal chordate lineage of amphioxus is ideally placed to understand the dynamics of the gene regulatory network evolution that produced these novelties. We report here the discovery that the cephalochordate lineage possesses two *Pax3/7* genes, *Pax3/7a* and *Pax3/7b*. The tandem duplication is ancestral to all extant amphioxus, occurring in both *Asymmetron* and *Branchiostoma*, but originated after the split from the lineage leading to vertebrates. The two paralogues are differentially expressed during embryonic development, particularly in neural and somitic tissues, suggesting distinct regulation. Our results have implications for the study of amphioxus regeneration, neural plate and crest evolution, and differential tandem paralogue evolution.

## Introduction

Susumu Ohno proposed in 1970^1^ that gene duplication might be an important evolutionary mechanism for generating diversity. The evolutionary fate of paralogues is influenced by both the mechanism of duplication and by the properties and functions of the genes involved, and various models have been developed to explain their adaptive trajectory^2,3^. Genes with a high degree of connectivity to regulatory regions and other gene products, and that are related to functions including development, neurogenesis, and organismal complexity, have been preferentially preserved following vertebrate whole genome duplications (WGDs). In contrast, functions primarily related to the immune response are over-represented among tandem/segmental duplications^4^. Because of the preferential survival of control genes such as transcription factors, duplications have had an important and complex influence on the evolution and expansion of gene regulatory networks (GRNs) (reviewed by Voordeckers *et al*.)^5^. Duplications of developmental control genes, and the opportunities for morphological novelty and complexity that they afford, are therefore important in the course of evolution.

The WGD events now thought to have occurred at the origin of vertebrates represent one such juncture, when the sudden genomic redundancy may have allowed the vertebrates to develop their synapomorphies^6^, specifically the head, neural crest, and neurogenic placodes^7^. The members of the GRN used to regulate the ontogenesis of the neural crest were mostly present in the chordate ancestor, but several were recruited from separate genetic pathways in vertebrates^8^, perhaps only possible because of the relaxation of genetic constraints afforded by WGD. Among the constituents of the ancestral neural patterning GRN is the neural plate border specification homeobox transcription factor *Pax3/7*^9^, which is present in vertebrates as the ohnologues *Pax3* and *Pax7*. These genes are necessary for neural crest induction^10,11^, and play later essential roles in neural crest cell migration, proliferation and differentiation (reviewed by Monsoro-Burq)^10^. *Pax3/7* genes also play an important role in somitogenesis and myogenesis, specifying primitive myogenic cells^12,13^, and later maintaining a population of quiescent muscle satellite cells^13^ that reactivate to perform muscle repair and regeneration^14,15^. Pax3/7 is believed to have ancient neurogenic^16^ and possible myogenic^17,18^ functions.

Vertebrate *Pax3* and *Pax7* have retained a high degree of sequence similarity, and possess similar but also non-redundant roles in somitogenesis and neural plate, tube, and crest development that diverge as development progresses^19,20^. They also participate in clade-specific hierarchies of interdependent regulation^21,22^. Their functions diverge more clearly in both embryonic and adult muscle development, with *Pax3* interfacing with 10-fold fewer transcription regulating sites than *Pax7*, with a comparatively much lower affinity for homeodomain motifs^23^, and conferring different properties to muscle satellite cells^24^. Thus, *Pax3* and *Pax7* illustrate how functional divergence in ohnologues following WGD may have contributed to the elaboration of vertebrate novelties and their diversification.

Comparative studies in cephalochordates, the invertebrate chordate sister group to Olfactores (tunicates and vertebrates), can provide important insight into the evolution of GRNs underlying vertebrate innovations. Unlike vertebrates, cephalochordates did not undergo whole genome duplication, have relatively ancestral-like genomes and possess conserved chordate morphology, including the presence of a dorsal hollow nerve cord, a notochord and segmented musculature (reviewed in Bertrand and Escriva)^25^. Previous research identified a single *Pax3/7* in the amphioxus *Branchiostoma floridae*, the embryonic expression of which broadly recapitulates that of vertebrate *Pax3* and *Pax7*^26^. *Pax3/7* expression has also been reported in adult muscle satellite-like cells in the amphioxus regenerative blastema^27^, a role that may be ancestral among bilaterians^17^.

Unexpectedly, we have discovered a divergent *Pax3/7* gene, unlike the known *Branchiostoma lanceolatum* or *B*. *floridae* orthologue^26^, but closely resembling the gene described in *B*. *belcheri*^28^. Previous studies had identified only a single copy of *Pax3/7* in cephalochordates, expressed in the neural plate border at the onset of neurulation, in somitogenesis, in the later development of the nervous system and larval musculature^26,29,30^ and in the adult segmental muscles^31^. We show here that the cephalochordate clade underwent a tandem duplication of the *Pax3/7* gene before the most recent common ancestor of extant cephalochordates, and that the two paralogues are differentially regulated in amphioxus development. Our discovery is a clear example of developmental control gene duplication and evolution in the context of a chordate genome untouched by WGD events.

## Results

### Cephalochordates possess two *Pax3/7* paralogues

**Figure 1:**
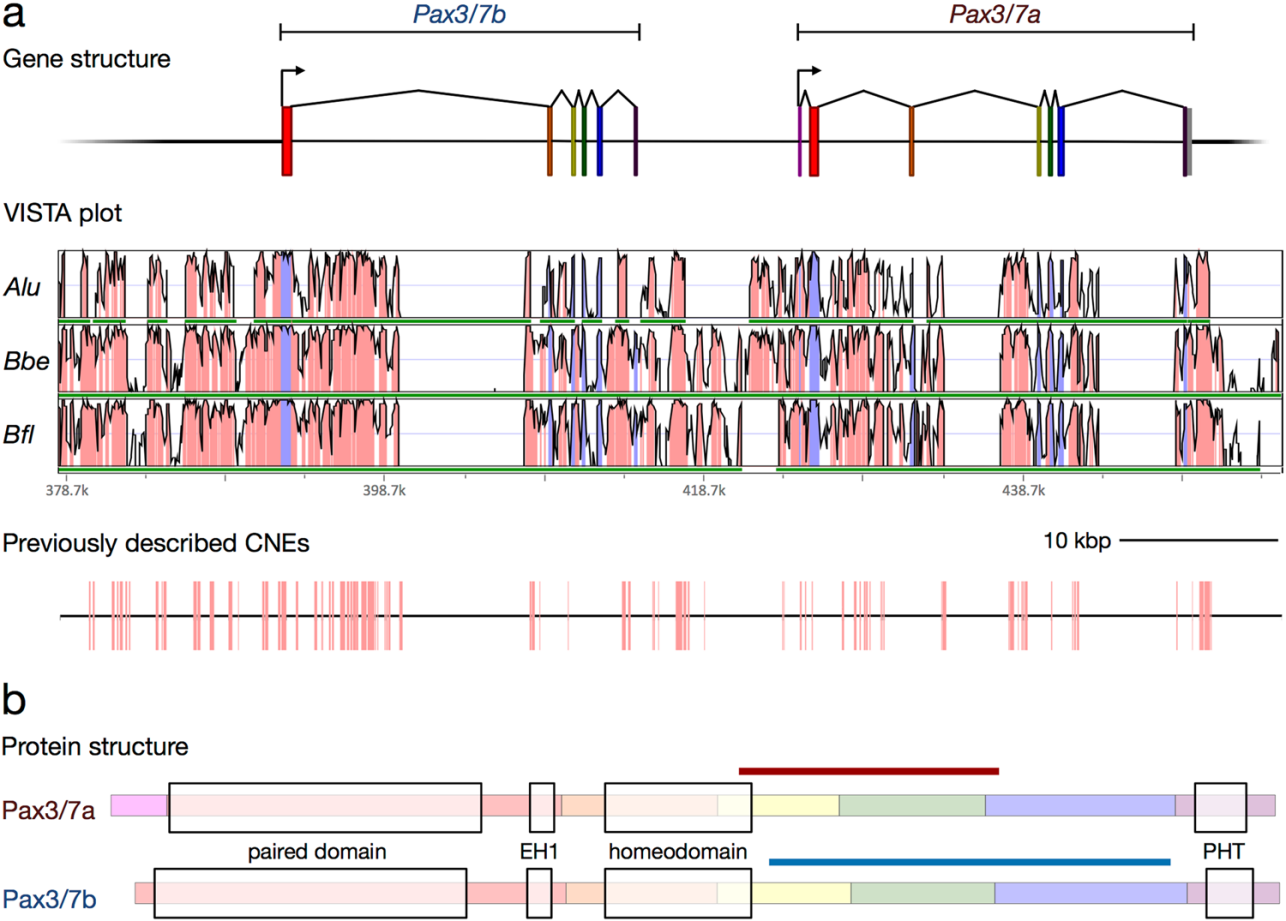
The structure and conservation of the cephalochordate *Pax3/7* gene pair. (**a**) Gene models (top), VISTA plot (middle) and map of previously described *B*. *floridae*/*A*. *lucayanum* CNEs^36^ (bottom) on the *Branchiostoma lanceolatum* scaffold. *Alu* = *A*. *lucayanum*; *Bbe* = *B*. *belcheri*; *Bfl* = *B*. *floridae*. In the VISTA plot, the horizontal axis indicates position on the *B*. *lanceolatum* genomic scaffold; green bars indicate coverage by the genome of the labelled species, and vertical axis indicates percent identity in a 45 bp rolling window, with a range of 50% to 100%. Pink colouration indicates regions exceeding the threshold of 90%, while blue indicates exonic sequence. Details of the scaffolds used in the VISTA analysis are reported in Supplementary Table S1. Scale bar = 10,000 base pairs. (**b**) Protein structure of the *Pax3/7* genes in amphioxus. Each exon is highlighted with a colour corresponding with its colour in (**a**). Conserved domains are indicated with light boxes; the paired domain, the EH1 domain (also known as the Octapeptide motif or TN), the homeodomain, and the Paired-type Homeodomain Tail^45^. The positions of the paralogue-specific probes on the transcripts are marked with a coloured bar. An annotated sequence alignment is presented in Supplementary Fig. S3.

While classifying the homeobox gene complement of a regenerative transcriptome of the European amphioxus *Branchiostoma lanceolatum*^32^, we identified a transcript that much more closely resembled the previously described *B*. *belcheri* *Pax3/7* homologue^28^ than either those of *B*. *lanceolatum* or *B*. *floridae*^26^. Exhaustive searches in the available *Branchiostoma* genomes indicated that *Branchiostoma* species possess two paralogues of *Pax3/7*, which we named in order of original discovery, *Pax3/7a* (described in 1999^26^ in *B*. *floridae*, and in 2008^33^ in *B*. *lanceolatum*) and *Pax3/7b* (described in 2005^28^ in *B*. *belcheri*). Construction of gene models revealed that the genes lie adjacent to one another in the genome and are separated by approximately 10 kbs. They are also in the same orientation, and share a similar exon and domain structure (Fig. 1), indicating that the paralogues are probably the result of a tandem gene duplication. We also found *Pax3/7a* and *Pax3/7b* in the *Asymmetron lucayanum* transcriptome^34^ and genome^35^, suggesting that the *Pax3/7* duplication event is likely to have occurred in the common ancestor of all extant cephalochordates.

### *Pax3/7b* has lost its first exon

Previous reconstructions of *Pax3/7a* seem to have inadvertently combined the 5′ end of the first *Pax3/7b* exon with the paralogous exon of *Pax3/7a*, probably due to the almost complete conservation of nucleotide identity between the two genes in the paired box domain. Also, the Paired box-containing exon in *Pax3/7a* does not have a start codon. However, transcriptomic data led us to identify a new *Pax3/7a* 5′ exon containing several potential start codons. Comparison with the exon structures of other deuterostome *Pax3/7* homologues indicates that the presence of an exon before the paired box-containing exon is probably the ancestral state (Supplementary Table S4), indicating that *Pax3/7b* has most likely lost the ancestral first exon.

### The cephalochordate *Pax3/7* locus is highly conserved

We aligned the relevant genomic scaffolds for *B*. *lanceolatum*, *B*. *floridae*, *B*. *belcheri*, and *A*. *lucayanum* in mVISTA (Fig. 1a), revealing high levels of conservation of non-coding sequence near the cephalochordate *Pax3/7* locus (≥90% identity over the majority of the ~74 kb window shown in Fig. 1). We identified 84 *B*. *floridae*/*A*. *lucayanum* pairs of CNEs (Conserved Non-coding Elements) previously described by Yue *et al*., Supplementary File 6^35^ within 20 kbs of the *B*. *lanceolatum* *Pax3/7* locus (Fig. 1a), covering about 12% of the non-coding sequence in this region. No CNE was found to reoccur in this window, implying divergence in the *cis*-regulatory landscape of the two paralogues.

### *Pax3/7a* and *b* are not direct orthologues to *Pax3* and *Pax7*

**Figure 2:**
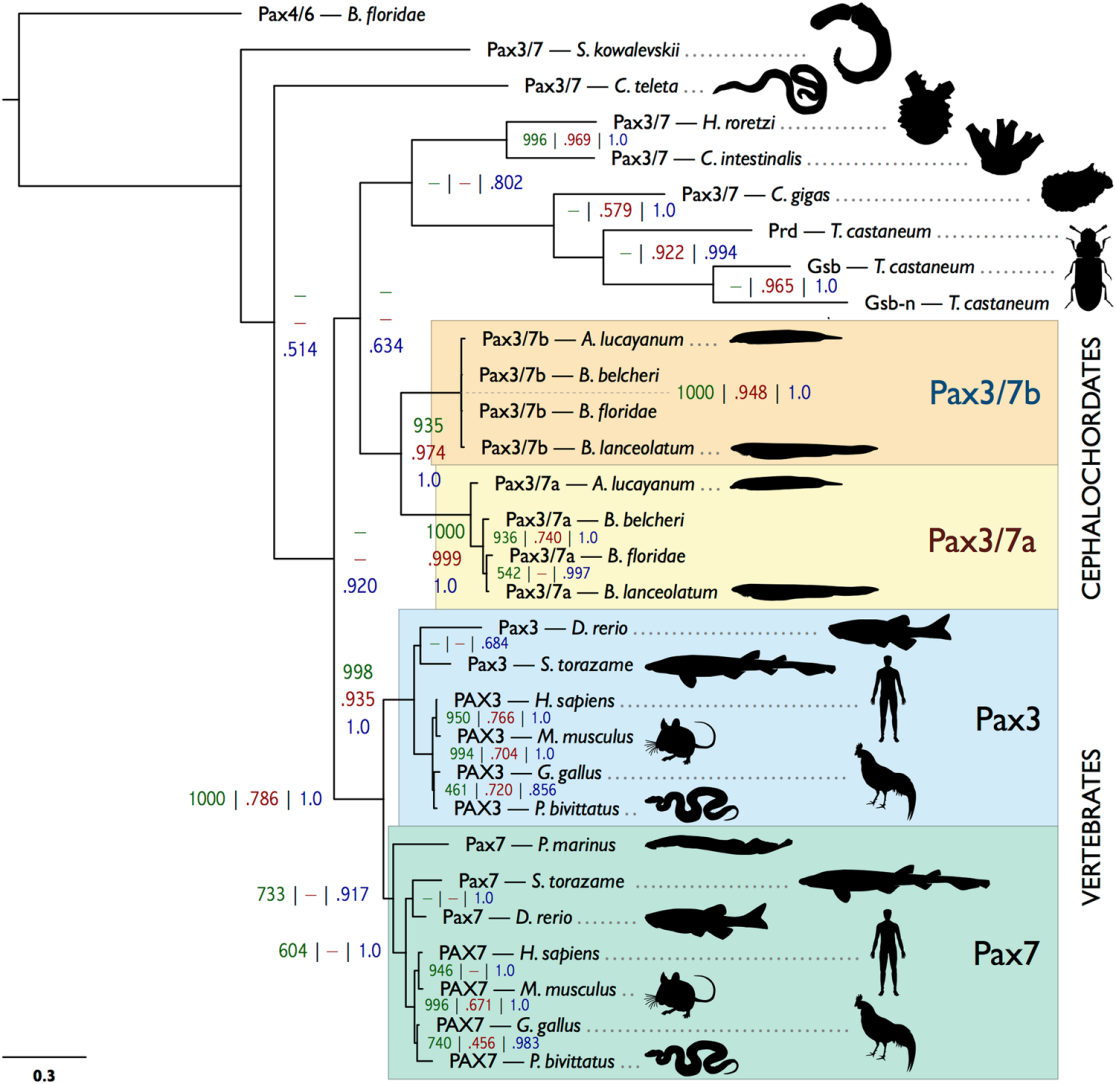
Bayesian tree of *Pax3/7* genes. Support values are presented as follows: bootstraps out of 1000 from PHYLIP (dark green) | bootstraps out of 1.0 from equivalent nodes from maximum likelihood (dark red) | posterior probabilities from equivalent nodes from a Bayesian analysis (dark blue). Absence of an equivalent node in the corresponding analysis is indicated by a dash. The accession numbers of all included sequences are reported in Supplementary Table S2 (Supplementary File 1). The scale bar in the lower left corner indicates amino acid substitutions per site. *B*. *floridae* = *Branchiostoma floridae*; *S*. *kowalevskii* = *Saccoglossus kowalevskii*; *C*. *teleta* = *Capitella teleta*; *H*. *roretzi* = *Halocynthia roretzi*; *C*. *intestinalis* = *Ciona intestinalis*; *C*. *gigas* = *Crassostrea gigas*; *T*. *castaneum* = *Tribolium castaneum*; *A*. *lucayanum* = *Asymmetron lucayanum*; *B*. *belcheri* = *Branchiostoma belcheri*; *B*. *lanceolatum* = *Branchiostoma lanceolatum*; *D*. *rerio* = *Danio rerio*; *S*. *torazame* = *Scyliorhinus torazame*; *H*. *sapiens* = *Homo sapiens*; *M*. *musculus* = *Mus musculus*; *G*. *gallus* = *Gallus gallus*; *P*. *bivitattus* = *Python bivitattus*; *P*. *marinus* = *Petromyzon marinus*.

We performed a phylogenetic analysis of a selection of available Pax3/7 family sequences from vertebrates, tunicates, cephalochordates, hemichordates, annelids, molluscs and insects (Fig. 2). Support values are formatted as number of neighbour joining bootstraps out of 1000, proportion of maximum likelihood bootstraps out of 1.0, and Bayesian posterior probability out of 1.0, separated by vertical bars. Our analysis produces strongly-supported cephalochordate-only clades containing Pax3/7a (1000 | 0.999 | 1.0), Pax3/7b (1000 | 0.948 | 1.0) and Pax3/7a + Pax3/7b (935 | 0.974 | 1.0). The vertebrate sequences group similarly; Pax3 (998 | 0.935 | 1.0), Pax7 (733 | − | 0.917) and Pax3 + Pax7 (1000 | 0.736 | 1.0). Despite the more ambiguous placement of the other Pax3/7 sequences included in the analysis, these strongly-supported clades corroborate the hypothesis that the cephalochordate *Pax3/7* duplication event was separate to that of the vertebrates, and that neither Pax3/7a nor Pax3/7b is a direct orthologue of either Pax3 or Pax7. The Pax3/7 sequences of tunicates (*H*. *roretzi* and *C*. *intestinalis*) and the non-chordate deuterostome sequence (*S*. *kowalevskii*) have diverged substantially, which is reflected in their phylogenetic distance from the chordate and cephalochordate genes. BLAST searches were performed in available echinoderm data, but, as in previous studies^36^ no Pax3/7 homologue was found.

### *Pax3/7* paralogues are differentially expressed during development

**Figure 3:**
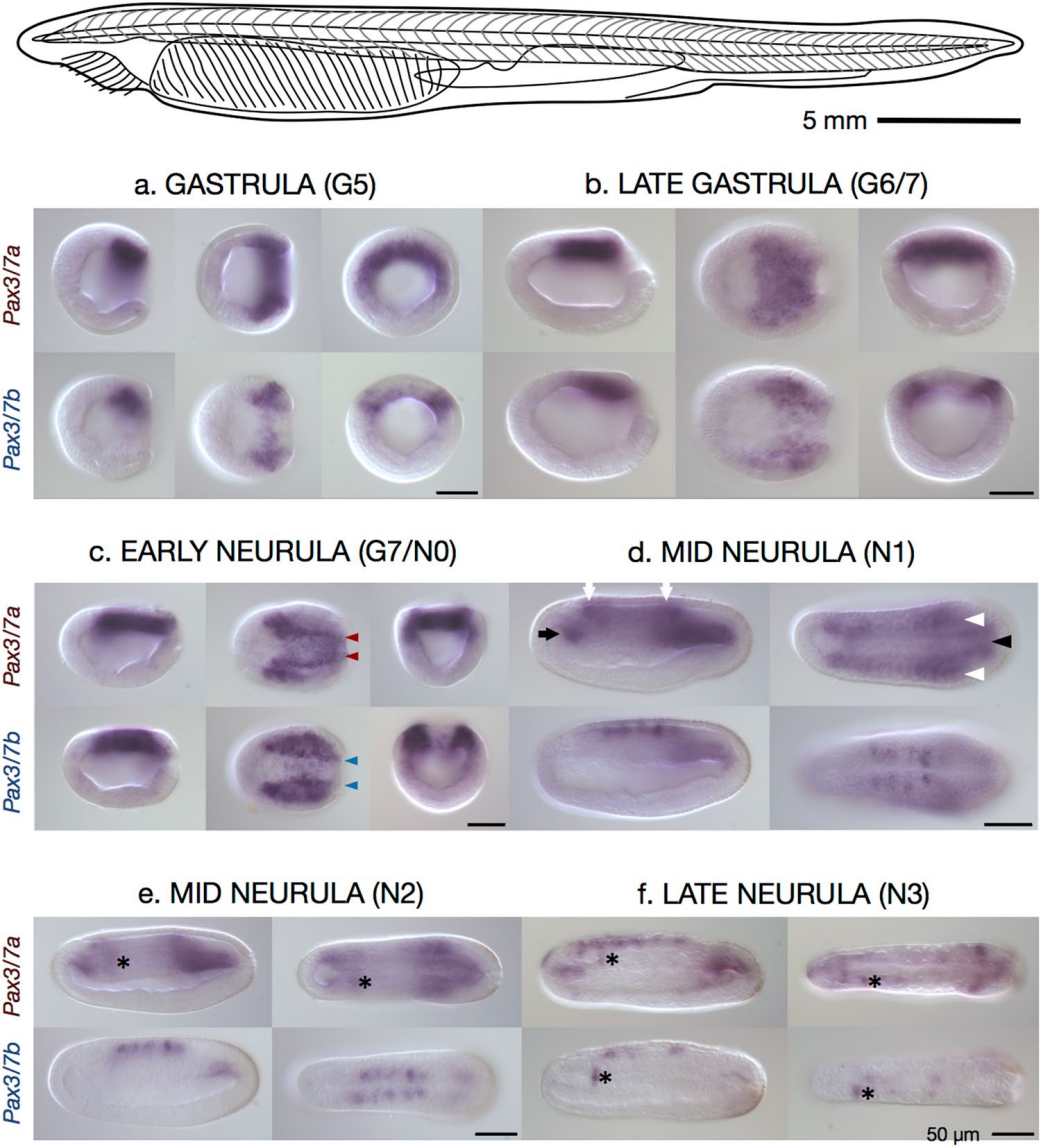
Expression of *Pax3/7a* and *Pax3/7b* in a *B*. *lanceolatum* early developmental time course. Top: Illustrative line drawing of adult *B*. *lanceolatum*. Scale bar ≈5 mm. Below: Whole mount *in situ* hybridisation images of *Pax3/7a*-specific probe (top row of each block) and *Pax3/7b*-specific probe (bottom row of each block) in *B*. *lanceolatum* embryos. Views are presented, in left-to-right order: lateral, dorsal, and blastoporal (gastrula and early neurula only). Lateral and dorsal views are oriented with the anterior to the left. **(a)** Gastrula, G5, 10 hours post fertilisation (hpf). **(b)** Late gastrula, G6/7, 12 hpf. **(c)** Early neurula, G7/N0, 14 hpf. (**d**,**e**) Mid neurulae: N1, 16 hpf and N2, 21 hpf. **(f)** Late neurula, N3, 24 hpf. Domains of expression are marked throughout as follows: coloured arrowheads — differentially patterned neural plate border expression; black arrow — expression in the anterior mesodermal tissue; white arrows — in the anterior end and posterior of the neural tube; white arrowheads — in the postero-lateral somitic tissue; black arrowhead — in the postero-medial notochord tissue; asterisk — (placed immediately posteriorly to) the sinistral domain of expression found in both paralogues and in *A*. *lucayanum*. Scale bars = 50 micrometres.

To visualise the expression of the two *Pax3/7* paralogues in early development, we performed whole mount *in situ* hybridisation on a time-course of *B*. *lanceolatum* embryos from mid-gastrula (G5) to L2 larvae (Fig. 3, Supplementary Fig. S1) and *A*. *lucayanum* embryos (Supplementary Fig. S2) using probes designed to target the divergent 3′ end of *Pax3/7a* and *Pax3/7b* transcripts (see Fig. 1, Supplementary Fig. S3). The *Pax3/7a* probe covered a region with 54.5% similarity (with 33 gaps) when aligned with MAFFT to *Pax3/7b*, and the Pax3/7b probe covered a region with 51.1% similarity (with 72 gaps) when aligned to Pax3/7a. Our results indicate that the paralogues are differentially expressed during embryonic development.

### Expression of *Pax3/7a*

In mid-gastrulae (G5, Fig. 3a), *Pax3/7a* is expressed in a semicircular band in the dorsal endoderm of the blastoporal lip. This expression pattern remains relatively diffuse in the late gastrula (G6/7, Fig. 3b), but by the early neurula (N0, Fig. 3c) the expression domain has become condensed into lines running symmetrically either side of the midline (Fig. 3c, red arrowheads) with enlarged anterior patches, though weak expression persists throughout the posterior. By the hatchling neurula (N1, Fig. 3d), *Pax3/7a* has diffuse expression with greater concentration in five indistinct, bilaterally symmetrical areas; the anterior mesodermal tissue (black arrow), the anterior end and posterior of the neural tube (white arrows), the postero-lateral somitic tissue (white arrowheads), and the postero-medial notochord tissue (black arrowhead). These expression domains continue with little change through to the mid neurula (N2, Fig. 3e), except for the appearance of a distinct domain of asymmetrical *Pax3/7a* expression in the anterior (marked throughout by an asterisk placed just posteriorly), which is consistently absent or very weak on the right side. In the late neurula (N3, Fig. 3f), *Pax3/7a* expression has become condensed into the anterior and posterior mesodermal regions, and into the left anterior somite. Patchy and granular neural regions of expression have also appeared. The asymmetrical domain persists into the early larva (L1, Supplementary Fig. S1a) while the other domains of expression are substantially reduced such that only a few anterior neural and the posterior mesodermal domains are present. This pattern continues in the L2 and L3 larvae (Supplementary Fig. S1b and c), with faint, patchy neural expression reappearing in the latter stage.

### Expression of *Pax3/7b*

In mid-gastrulae (G5, Fig. 3a), *Pax3/7b* is expressed in smaller lateral patches in the dorsum in both germ layers. This lateral expression pattern continues in the late gastrula (G6/7, Fig. 3b); by the early neurula (N0, Fig. 3c) the interior lateral borders of the expression domain have become strongly resolved (Fig. 3c, blue arrowheads), though weak medial expression continues. *Pax3/7b* expression overlaps with *Pax3/7a* in the posterior regions but with a much weaker signal. By the N1 stage (Fig. 3d), in contrast to *Pax3/7a*, there are five distinct, symmetrical domains of *Pax3/7b* expression in the dorsolateral neural tube. These spots are flanked at their anterior and posterior limits by the weaker, more diffuse regions of *Pax3/7a* expression (white arrows). This expression pattern continues with little change through to the mid neurula (N2, Fig. 3e). By stage N3 (Fig. 3f), the neural regions of expression are reduced in size and number, retaining only the two anterior-most and posterior-most spots, while the strong asymmetrical domain of expression in the left anterior somite previously distinguished by *Pax3/7a* expression is now also labeled by *Pax3/7b* (asterisks). This domain persists with strong expression into the early larva (L1, Supplementary Fig. S1a) while expression ceases elsewhere in the L2 and L3 larvae (Supplementary Fig. S1b and c).

## Discussion

Gene duplication is an important mechanism in evolution, providing a potent source of new genetic material on which evolution can act outside the constraints on single-copy genes. Transcription factors stand out as a particularly important subset of retained and adapted paralogous genes. Paralogue divergence includes subfunctionalisation and neofunctionalisation of binding specificity and motif recognition, upstream regulatory control, and cofactor interaction, which all provide opportunities for more intricate spatiotemporal expression control and the potential for the generation of novel gene regulatory networks and morphology^5^.

The two rounds of whole genome duplication (2R-WGD) at the base of the vertebrate lineage^37^ provided an ample source of stoichiometrically-balanced raw genetic material, possibly facilitating the elaboration of vertebrate novelties including the head, neural crest, and neurogenic placodes^6,7^. In contrast, cephalochordate genomes bear no indications of paleopolyploidy events^37–39^, and share more similarities in terms of architecture and gene content with the chordate ancestral genome than other extant chordate clades^40^. Cephalochordates therefore have many fewer paralogues than vertebrates, though both RNA-mediated and DNA-mediated duplications have been described. Among the latter, homeobox genes are most numerous; paralogues have been found in *Evx*^41,42^, *Emx*^41,43,44^, *Mnx*, *Vent*, *Nk1*, *Nedx*, *Uncx*, *Lhx2/9*, *Irx*, *Pou3*^44^, and *Hox9-15*^39,45^, many of which are the result of small-scale tandem duplications. Of these, only *Vent1* and *Vent2* have been the subject of detailed functional assays, which established their *cis-* and *trans-*regulation in the amphioxus dorsoventral patterning regulatory network^46^ and their expression in pharmacologically manipulated embryos^47^.

Our data from three species of *Branchiostoma* and *Asymmetron lucayanum*, a representative of the earliest branching of the extant amphioxus genera, support the idea that tandem gene duplication may have been an important mechanism for generating cell type diversity in the cephalochordate ancestor. We report that amphioxus possess two paralogues of *Pax3/7*, a gene notable for its functions in neural plate border specification, its vertebrate roles in neural crest and placode specification, and for its involvement in somitogenesis, myogenesis and the population of regenerative muscle satellite cells possibly common to all bilaterians^17^. We confirm that this duplication predates the modern cephalochordate radiation but post-dates the divergence from other chordates, implying that the chordate ancestor had a single copy.

One of our key findings is that *Pax3/7a* and *Pax3/7b* diverged symmetrically but heterogeneously between duplication and the cephalochordate radiation (Fig. 2). They share very strong nucleotide sequence conservation and 100% amino acid sequence identity in the paired domain, EH1/Octapeptide motif and homeodomain, possibly the result of gene conversion. In contrast, they have diverged substantially in the linker regions, the N-terminus (where *Pax3/7b* seems to have lost an exon) and the four exons of the C-terminus. The paralogues have changed little since their divergence, both in coding sequence and local CNEs; of the pair, *Pax3/7a* has changed more since the *Asymmetron*/*Branchiostoma* speciation events, indicating it might be under slightly relaxed selection, but has a more prototypical PHT domain^48^ (Supplementary Fig. S3; Supplementary File 3, residues 447–467 at positions 801–829), while *Pax3/7b* is more conserved among species. Pronounced evolutionary asymmetry is common amongst tandem paralogues (reviewed by Holland *et al*.)^49^, for instance, in *AmphiEvx*; however, examples in which asymmetry is not observed have also been documented (*AmphiEmx*).

Although cephalochordates are considered to be slow-evolving, the pattern we observe in paralogue divergence is also consistent with the recent estimate that the crown cephalochordate node dates to only 38.8–46.0 million years ago (MYA), in contrast to previous results placing it ~120–250 MYA (see Igawa *et al*.^50^ and references therein). Based on their calibration date of the cephalochordate/Olfactores split approximately 550 MYA, the duplication, fixation, fate-determination and preservation phases of paralogue evolution (see Innan and Kondrashov)^51^ all occurred in the ~500 MYA interval during which no evident radiation occurred. Comparatively rapid change and quicker preservation is considered typical of tandem duplications^5^, although as *Pax3/7* genes are transcription factors involved in development, and specifically neurogenesis^52^, their sequence and expression domain change may have been severely constrained.

Symmetry of sequence evolution rate between paralogues is considered indicative of subfunctionalisation^53^. The evolutionary trajectory of cephalochordate *Pax3/7* duplicates, based on the symmetry of sequence change evident in Fig. 2, seems to accord with the duplication-degeneration-complementation (DDC) model of Force *et al*.^3^ or the specialisation model of Hughes^2^. According to these models, the duplicated pair, under relaxed purifying selection, accumulates either mutations that complementarily degrade (DDC) or improve (specialisation) their capacity to perform subsets of their pre-duplication function, until the loss of either paralogue is deleterious. The only non-duplicated chordate or deuterostome outgroups for ancestral *Pax3/7* function are found in the tunicates and hemichordates. However, both groups have a highly divergent *Pax3/7* sequence, and the former of which has a very derived genome and morphology. Consequently, it is difficult to determine the exact set of ancestral functions of the *Pax3/7* pro-orthologue in the chordate ancestor. Nevertheless, a conserved role in neural border specification is highly probable, given enrichment of *Pax3/7* in lateral neuroblasts in a number of bilaterians^9^.

Although the DNA-binding domains of *Pax3/7a* and *Pax3/7b* are identical, it is likely that the differences in the C-terminus and in the linker regions between the conserved domains are sufficient to alter their functionality. Amino-terminal sequence changes have been shown to affect the binding specificity of DNA-binding domains and homeodomains in general^18,54^ and Pax genes specifically (reviewed by Mayran *et al*.)^52^.

Small sequence changes have the potential to differentially modify the binding affinity of the paired domain and homeodomain, the binding modality of the paired subdomains, and subnuclear localisation^56^. The modest differences between Pax3 and Pax7 sequence, located mostly in the C-terminus, are enough to produce substantial differences in target activation in myogenesis^23^ (Mayran *et al*.^55^ and references therein). The extent of these substantial functional effects caused by the minor differences in mutants, splice variants and between vertebrate *Pax3* and *Pax7* is an indication that *Pax3/7a* and *Pax3/7b*, which have diverged more than *Pax3*/*Pax7*, probably behave differently with regard to target recognition and interaction with cofactors. Such sequence change has been highlighted as an important but under-appreciated mechanism in the evolution of developmental GRNs^57^.

Regardless of putative differences in downstream activity, *Pax3/7a* and *Pax3/7b* are expressed differently during gastrulation and neurulation in *B*. *lanceolatum*, demonstrating that the paralogues have diverged in their *cis-*regulation. *Pax3/7a* and *Pax3/7b* are expressed in partially overlapping but distinct domains in the neural plate (G5 to N0, Fig. 3a–c, red and blue arrowheads), presumably as the result of modification of an ancestral neural plate domain. *Pax3/7a* is expressed throughout the dorso-posterior mesoderm prior to neurulation (G5 and G6/7, Fig. 3a,b) while *Pax3/7b* is restricted to smaller, bilaterally symmetrical dorso-posterior regions in both the mesoderm and ectoderm, consistent with a role in the initial specification of the neural plate border. Distinct lateral lines of expression do appear in *Pax3/7a* in the late gastrula/early neurula (G7/N0, Fig. 3c), but diffuse expression remains throughout the posterior. By the mid-neurula, the paralogues seem to have switched to a different expression programme, one in which their expression patterns have the least overlap. Particularly notable are the tight, defined neural spots of *Pax3/7b* and the appearance of the asymmetrical, sinistral domain (the anterior somite^26^) of expression that first appears in *Pax3/7a* (left of asterisk throughout, N2, Fig. 3e) and later appears in *Pax3/7b* (N3, Fig. 3f). As the embryo becomes a larva, the two expression patterns converge until both expression patterns are largely restricted to the asymmetrical domain (L1 and 2, Supplementary Fig. S1a–c). Thus, divergence between duplicate expression patterns increases during gastrulation and early neurulation, peaking at mid-neurula stages, consistent with function partitioning. Although we still know very little about *Asymmetron lucayanum* developmental gene expression, our data indicate similar results for *Pax3/7* paralogues in this species (Supplementary Fig. S2). This is currently the only example in cephalochordates in which a gene duplication event has been shown to predate the divergence of extant lineages and for which expression data exist in more than one genus.

Our results broadly recapitulate previous *Pax3/*7 expression data from *B*. *lanceolatum* (Fig. 3H,I and J of Somorjai *et al*.)^33^, considering that the latter used a probe with probable cross-reactivity between the 5′ conserved region of *Pax3/7a* and *Pax3/7b*. In contrast, the *B*. *lanceolatum* expression patterns are not a perfect subset of the *Pax3/7*(*a*) domains reported for *B*. *floridae* (Fig. 5 of Holland *et al*.)^26^, who used a similarly cross-reactive probe. Potentially missing from our patterns are the anterior somitic and mesodermal expression (Fig. 5F,G,I and K of Holland *et al*.)^26^, the distinct anterior neural spot (arrow, Fig. 5K,M,P and Q of Holland *et al*.)^26^ and the larval axial musculature and notochord expression (Fig. 5M,P, and Q of Holland *et al*.)^26^. Minor discrepancies are not unusual, but significant differences among *Branchiostoma* species are rare^33^. It is possible that these differences are caused by the general variability between probes for the same target, *Pax* gene probe cross-reactivity, or experimental sensitivity. The probes we used were by necessity relatively short in order to limit possible cross-reaction of highly conserved regions, but the expression patterns we observed are highly specific and reproducible, suggesting they reflect the core domains of *Pax3/7a* and *Pax3/7b*.

In contrast to what we see in amphioxus, differences between vertebrate *Pax3* and *Pax7* early developmental expression are much less pronounced, to the extent that they have ‘swapped’ expression profiles during evolution (see Monsoro-Burq^10^, and references therein). *Pax3/Pax7* appear in the neural plate border during neural induction in the early gastrula, and intensify at the lateral edges to mark the dorsal edge of the closing neural tube, a pattern comparable to late gastrula/early neurula expression in amphioxus. *Pax3* and/or *Pax7* are also expressed throughout the posterior dorsal neuraxis, an approximate analogue of the neural spots in *Pax3/7b* and later *Pax3/7a*, though these spots are more spatiotemporally restricted.

While *Pax3* and *Pax7* appear to play semi-redundant roles in neural development, they diverge in function in vertebrate myogenesis (reviewed by Buckingham & Relaix)^58^. *Pax3* acts broadly from the onset of myogenesis in the presomitic mesoderm to the dermomyotome, while *Pax7* expression is later and restricted to a dermomyotomal subdomain. These PAX3/PAX7 positive cells form a proliferative muscle progenitor population that eventually positions itself underneath the basal lamina on the muscle fibres. In the adult, these cells become a heterogenous population of quiescent satellite cells; all are maintained by *Pax7* expression, but some also expresses *Pax3*, which is known in this context to be an inadequate substitute, binding 10-fold fewer targets, most of which are also targets of PAX7. During myogenesis, *Pax3* and *Pax7* seem to be responsible for maintaining the cells in a proliferative/quiescent but undifferentiated state. Lack or cessation of *Pax3* or *Pax7* expression in a cell can lead to apoptosis or cell cycle exit and muscle differentiation via MyoD, depending on the precise context.

Although the later myogenic roles of amphioxus *Pax3/7* genes are yet to be thoroughly characterised, at least one of the paralogues is known to be expressed in adult muscle, as *Pax3/7b* has been amplified from adult *B*. *belcheri* segmental muscle^31^. Whether both paralogues are involved in adult muscle development redundantly, or rather show temporal or tissue-specific patterns of expression (similarly to *Pax3* and *Pax7* in post-embryonic muscle development and regeneration in mice) is still unclear. Our initial identification of *Pax3/7b* transcripts in a tail blastema transcriptome clearly identifies a role in the adult regeneration process. However, previous characterization of *Pax3/7* in a population of satellite-like cells and the nerve cord during tail regeneration utilized a cross-reactive *in situ* hybridisation probe^27^. We therefore cannot currently rule out changes in paralogue function during postembryonic processes in amphioxus. Future studies are required to determine to what extent divergence has occurred in expression, downstream targets, and interaction with co-factors in both myogenic and neural contexts.

Amphioxus *Pax3/7* has been considered a useful proxy for understanding the properties and deployment of the chordate proto-*Pax3/7*. Our findings showing independent vertebrate and cephalochordate *Pax3/7* duplications – and the resulting functional and regulatory divergence – offer new insight into genomic constraint/plasticity, and evolvability of gene duplicates and GRNs in different duplication contexts. In amphioxus, tandem duplication and divergence of *Pax3/7* has resulted in a subfunctionalisation (and possibly neofunctionalisation) of ancestral neural plate border^9^ and muscle-related^17,18^ functions, many of which parallel those seen in vertebrate *Pax3* and *Pax7* following WGD. Dissecting the regulatory landscape of *Pax3/7* genes in amphioxus, including the function of the CNEs partitioned between paralogues, should shed further light on genome architecture evolution in chordates.

## Conclusions

We show that cephalochordates, which are considered to be a significant outgroup to vertebrates in the study of the evolution of the neural crest GRN, have two *Pax3/7* paralogues where it was previously thought that this family was represented by a single-copy gene in these animals. This discovery has implications both for previous and future studies of amphioxus development and regeneration, and for vertebrate studies in which cephalochordates are used as an outgroup. The amphioxus *Pax3/7* gene pair also offers a tantalising and tractable example of *cis*-regulatory and sequence subfunctionalisation after tandem duplication of a developmental transcription factor involved in the development of key chordate features.

## Methods

### Genomic & transcriptomic analysis

A tBLASTn^59^ search of a transcriptome, generated from the pre-amputation and blastemal tissues of a regenerating *B*. *lanceolatum* post-anal tail (14 dpa/stage 2 *sensu* Somorjai *et al*.)^27^ assembled with developmental transcriptomic data from Oulion *et al*.^60^, for homeodomains selected from HomeoDB^61^ retrieved a partial *Pax3/7b* sequence. Subsequent identification and comparison was done by alignment in Jalview 2.x^62^. The exon structures of *Pax3/7a* and *Pax3/7b* were manually predicted with reference to tBLASTn searches of the known sequences against the available genomes: the *B*. *lanceolatum* draft assembly (Bl71nemr) (European Amphioxus Genome Consortium), the *B*. *floridae* reference genome version 2.0^37^, the *B*. *belcheri* draft assembly^38^ (HapV2), and the *A*. *lucayanum* draft assembly^35^, and used to manually produce diagrams of the gene and protein structure, in reference to domains predicted by the Conserved Domain Database^63^ and the *Pax* gene conserved regions identified by Vorobyov & Horst^48^.

Transcriptomic support for both cephalochordate *Pax3/7* paralogues was obtained using tBLASTn and MEGABLAST searches of the *A*. *lucayanum* transcriptome^34^, *B*. *floridae* cDNA library^29^, a *B*. *lanceolatum* SRA (BioProject: PRJNA285432) and the unpublished regenerative transcriptome.

### Visualisation

Curated genomic sequences from *B*. *lanceolatum*, *B*. *floridae*, *B*. *belcheri* and *A*. *lucayanum* were uploaded to the web interface for mVISTA^64^ along with manually predicted annotations of the *B*. *lanceolatum* scaffold. These were aligned using the AVID alignment algorithm^65^ and the alignment was visualised with 45 bp calculation window, 45 bp minimum conserved width, and 90% conserved identity threshold parameters. Full details of the scaffolds and curation are presented in Supplementary Table S1.

The region of the *B*. *lanceolatum* genome represented in the VISTA plot was used as a query for a BLASTn search against the Conserved Non-Coding Elements database presented in Supplementary File 6 of Yue *et al*.^35^. Matching sequences were retrieved from the CNE database, and the sequences aligned back to the query using MAFFT–addfragments mode^66^. Spreadsheet tools were used to extract positional information and to generate the visualisation of distribution.

Exon positions for various deuterostome *Pax3/7* genes were extracted from their NCBI records listed under the accession numbers in Supplementary Table S4; protein domain positions were predicted using the Conserved Domain Database^63^ and manually corrected where homeodomain prediction was too short.

### Phylogenetic analysis

Protein sequences were predicted from the genomes of *B*. *lanceolatum*, *B*. *floridae*, *B*. *belcheri*, and *A*. *lucayanum* with reference to the published *B*. *floridae* Pax3/7a (EEN66816.1) and *B*. *belcheri* Pax3/7b (ABK54280.1) sequences. Gaps in the *A*. *lucayanum* gene models due to incomplete coverage were partially filled by manual assembly of the results of a tBLASTn search of the *B*. *lanceolatum* Pax3/7a and b protein sequences against the *A*. *lucayanum* SRA archive (SRR1138336). Complete coverage of *A*. *lucayanum* Pax3/7b was not possible.

Protein sequences for *Homo sapiens*, *Mus musculus*, *Gallus gallus*, *Python bivittatus*, *Danio rerio*, and *Scyliorhinus torazame* Pax3 and Pax7; *Petromyzon marinus* Pax7; *Halocynthia roretzi*, *Saccoglossus kowalevskii*, *Crassostrea gigas*, and *Capitella teleta* Pax3/7; and *Tribolium castaneum* Paired, Gooseberry, and Gooseberry-neuro were retrieved from the NCBI; all accession numbers for the phylogeny are reported in Supplementary Table S3. Sequences were aligned in Jalview using the MAFFT alignment algorithm with default settings^66^ and manually corrected.

Model selection was performed in ModelGenerator v0.85^67^ using default settings and 4 gamma categories. The model recommended (JTT + G + F) or its closest possible equivalent was selected in all subsequent phylogenetic analyses. A neighbour-joining analysis was performed in PHYLIP 3.69^68^, a maximum-likelihood analysis in MEGA-CC^69^, and a Bayesian analysis on the CIPRES Science Gateway^70^, using MrBayes 3.2.6^71^ on XSEDE. Full details of settings used in the analysis are presented in the Supplementary Note. The support values for equivalent nodes between analyses were mapped using the Python script in Supplementary File 3 onto the Bayesian tree and the consensus tree output was visualised in FigTree 1.4.2.

### Embryo collection

Adult European amphioxus (*Branchiostoma lanceolatum*) were collected from Argeles-sur-mer (France), kept in a semi-closed circulating system at 16.5 °C, and induced to spawn as described previously^72^. Populations of *A*. *lucayanum* were collected from Bimini (Bahamas), kept in filtered seawater at 25 °C, and induced to spawn as described previously^35^. Embryos for *in situ* hybridisation were fixed at relevant time points in fresh 4% PFA in MOPS salts (0.1 M MOPS, 2 mM MgSO4, 1 mM EGTA, & 0.5 M NaCl), transferred into 70% Ethanol and stored at −20 °C. *Branchiostoma lanceolatum* embryos were staged according to modifications suggested by Zhang *et al*.^73^.

### Cloning and probe synthesis

RNA was extracted from *B*. *lanceolatum* embryos fixed at a selection of developmental stages using TRIsure (Bioline) using the supplier’s protocol. *A*. *lucayanum* embryos fixed in RNAlater (Invitrogen) were transferred to TRIsure and treated similarly. cDNA libraries were produced using the Tetro cDNA Synthesis kit (Bioline). Gene fragments for probe synthesis were amplified by PCR using gene-specific primers (Supplementary Table S2) designed using *B*. *lanceolatum* transcriptomic (see above) and genomic sequences from *B*. *lanceolatum* and *A*. *lucayanum* cDNA. The amplicons were ligated into pGEM-T Easy vector (Promega) and transformed into the XL10-Gold (Stratagene) competent E. coli cell strain using standard heat shock protocols. Selected clones were cultured and extracted using peqGOLD (Peqlab) or Promega plasmid miniprep kits and sequenced for verification using Universal M13F (5′-GTAAACGACGGCCAGT-3′) and M13R (5′-AACAGCTATGACCATG-3′) primers at the University of Oxford Zoology Sequencing Service. Probe template was produced using PCR with M13 primers. Bands were verified using agarose electrophoresis and precipitated using sodium acetate (3 M, pH 5.2) and ethanol. DIG-labelled (Roche) antisense probes were transcribed *in vitro* using T3 and SP6 enzymes as appropriate, following standard protocols.

### *In situ* hybridisation

Whole mount *in situ* hybridisation was performed as previously reported^33^. In brief, embryos fixed in 4% paraformaldehyde and stored at −20 °C were rehydrated in NaPBS + 0.1% Tween and permeabilised with proteinase K, followed by post-fixation and acetic anhydride treatment to reduce background. A paralogue- and species-specific DIG-labelled probe was hybridised overnight at 65 °C to target mRNA in the embryos. Excess probe was washed back out through decreasing concentrations of formamide before being treated with RNAses to reduce background. The embryos were blocked against non-specific antibody binding and exposed to alkaline phosphatase-associated anti-DIG antibodies overnight at 4 °C. The embryos were finally stepped into buffer and NBT and BCIP (alkaline phosphatase substrates) introduced.

### Equipment and settings

To capture the images used in Fig. 3 and Supplementary Figs S1 and S2, embryos were mounted in 95% glycerol/5% PBS and examined under a Leitz DMRB microscope (Leica Microsystems) with Nomarski optics. Images were captured using a Retiga 2000R camera in the QCapture software suite (QImaging) and processed in the GNU Image Manipulation Package (GIMP) and Inkscape.

### Ethics statement

No specific permits were required for collection of animals used in this study. All procedures were in compliance with regulations for the experimental use of non-cephalopod invertebrates in the UK and EU (DIRECTIVE 2010/63/EU OF THE EUROPEAN PARLIAMENT AND OF THE COUNCIL of 22 September 2010 on the protection of animals used for scientific purposes).

### Statement of data availability

Full sequences for the genes described in the current study are available from the NCBI database (accession numbers in Supplementary Table S3).

## Electronic supplementary material

Supplementary Files, Tables and Notes
