# Supplementary material for: Pax3/7 duplicated and diverged independently in amphioxus, the basal chordate lineage
Source: Sci Rep. 2018 Jun 20;8:9414. doi: 10.1038/s41598-018-27700-x (PMC6010424; doi:10.1038/s41598-018-27700-x)
Supplement: Supplementary file 1 — Supplementary Files, Tables and Notes [file 41598_2018_27700_MOESM1_ESM.pdf]

Supplementary Files, Tables & Notes

Barton-Owen, Ferrier & Somorjai

Pax3/7 duplicated and diverged independently in amphioxus, the basal chordate lineage

Table of contents

Item	Description	Page #
**Supplementary Fig. S1**	WMISH images for *B. lanceolatum* L1 & L2	2
**Supplementary Fig. S2**	WMISH images for *A. lucayanum*	2
**Supplementary Fig. S3**	Annotated amino acid alignment of cephalochordate Pax3/7 sequences	3
**Supplementary Table S1**	Details of cephalochordate genomic data used in Fig. 1A (mVISTA plot).	4
**Supplementary Table S2**	Details of primers and amplicons used as probes in WMISH experiment (Figs. 3, 4, S1).	5
**Supplementary Table S3**	Details of accession numbers used in alignment/phylogenetic analyses.	6
**Supplementary Table S4**	Details of exon/intron structure for a selection of Pax3/7 homologues, showing the conserved exon structure.	7
**Supplementary File 2**	FASTA format alignment of bilaterian Pax3/7 genes	8-12
**Supplementary File 3**	FASTA format alignment of cephalochordate Pax3/7 genes	3
**Supplementary File 4**	Python script to map equivalent support values between unadorned .nwk format trees	14-16
**Supplementary Note**	Settings for the phylogenetic analyses	17-18

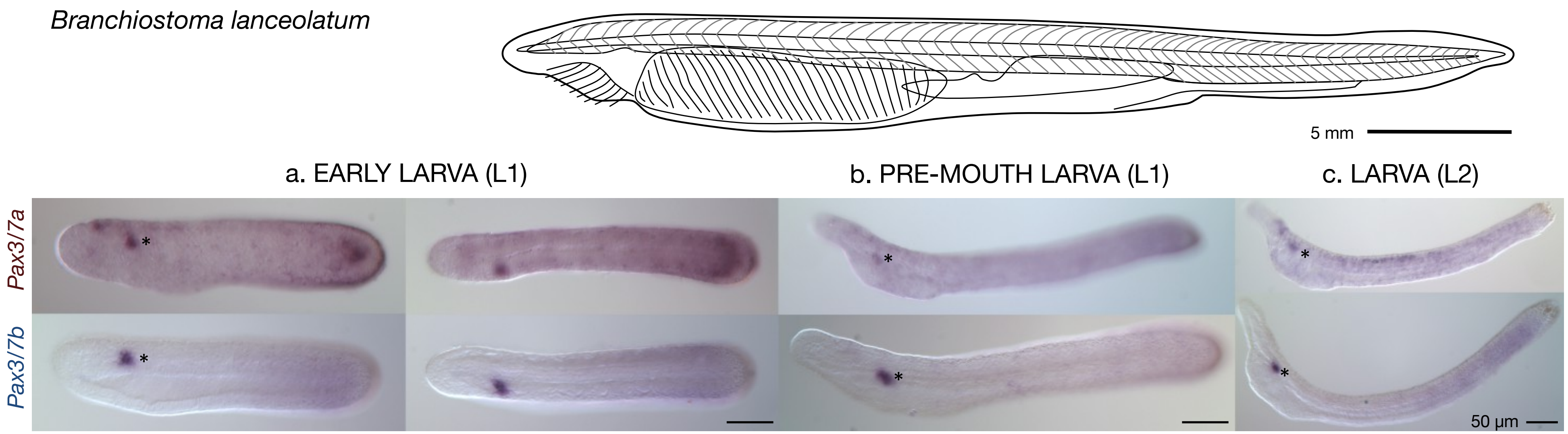

Supplementary figure S1: **Expression of Pax3/7a and Pax3/7b in a *B. lanceolatum* later developmental time course.** Top: Illustrative line drawing of adult B.*lanceolatum.* Scale bar ≈ 5 mm. Bottom: Whole mount *in situ* hybridization images of *Pax3/7a*-specific probe (top row of block) and *Pax3/7b*-specific probe (bottom row of block) in *B. lanceolatum* larvae. Views are presented in the left-to-right order: lateral (all) and dorsal (early larva only). All views are oriented with the anterior to the left. Asterisks mark to the immediate anterior the common sinistral domain of expression. (**a**) Early larva, L1, 30 hpf. (**b**) Pre-mouth neurula, L1, 36 hpf. (**c**) Larva, L2, 48 hpf. Scale bars = 50 micrometres.

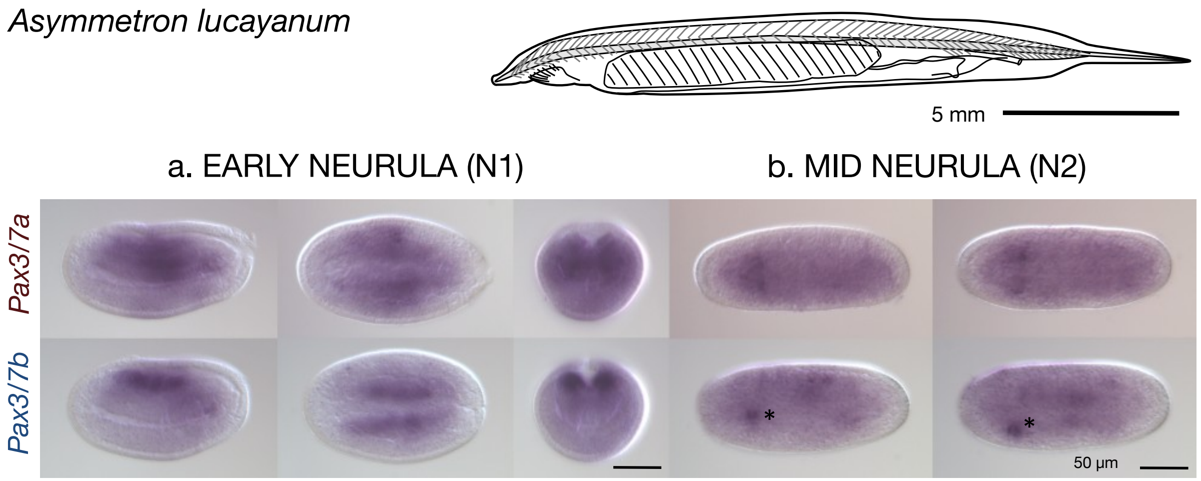

Supplementary figure S2: **Expression of *Pax3/7a* and *Pax3/7b* in an *A. lucayanum* early developmental time course.** Top: Illustrative line drawing of adult *A. lucayanum*, adapted from Andrews, E.A. *An undescribed acraniate:* Asymmetron lucayanum (Baltimore, 1893). Scale bar ≈ 5 mm. Bottom: Whole mount *in situ* hybridisation images of *Pax3/7a*-specific probe (top row of block) and *Pax3/7b*-specific probe (bottom row of block) in *A. lucayanum* embryos. Views are presented in the right-to-left order: lateral, dorsal, and blastoporal (early neurula only). Lateral and dorsal views are oriented with the anterior to the left. **(a)** Early neurula, N1, 12 hpf. **(b)** Mid neurula, N2, 16 hpf. Asterisks mark to the immediate anterior the sinistral domain of expression found in both paralogues and in *A. lucayanum*. Scale bars = 50 micrometres.

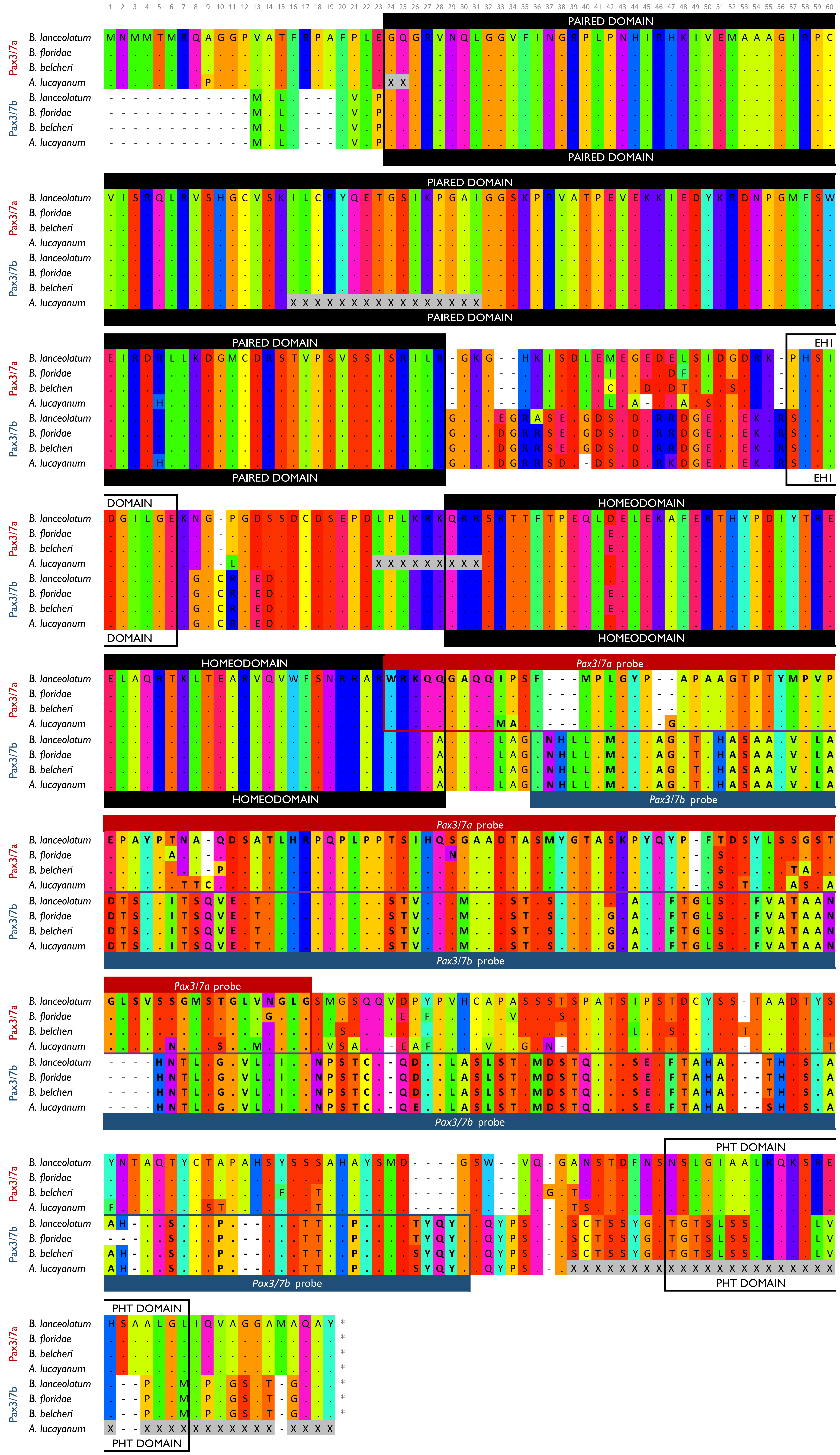

Supplementary Figure S3: Annotated amino acid alignment of cephalochordate Pax3/7 sequences. Residues are coloured according to the Taylor colour scheme. Domains are highlighted in white and black boxes, and the region covered by the probes highlighted by a red box (*Pax3/7a* probe) and a blue box (*Pax3/7b* probe).

Assembly	Scaffold/contig	Modifications
Bl71nemr	Sc0000222	None
BRAFL v2	scaffold23 contig4522	None
	scaffold23 contig 4524	None
HapV3	scaffold5	Trimmed to bases 2035000-4035000
GCA_001663935.1_Asyluc0.1		
	LZCU01018971.1_scaffold1268824_cov58	None
	LZCU01148733.1_scaffold328746_cov58	None
	LZCU01080238.1_scaffold2528110_cov53	Reverse complement
	LZCU01117694.1_scaffold2608874_cov71	Reverse complement
	LZCU01137041.1_scaffold2697051_cov71	Reverse complement; padded 3' & 5' with 2,000 Ns to prevent VISTA alignment ignoring it
	LZCU01078009.1_scaffold2501631_cov61	None
	LZCU01058212.1_scaffold20007_cov45	Reverse complement; extended 3' with SRA data
	LZCU01078009.1_scaffold2501631_cov61	Extended 5' with SRA data
	LZCU01179060.1_scaffold612678_cov42	Reverse complement; split into two pieces to allow insertion of scaffold628499; assembly gaps in first half improved with SRA data
	LZCU01180542.1_scaffold628499_cov66	Reverse complement
	LZCU01040462.1_scaffold1632309_cov71	None
	LZCU01174809.1_scaffold568960_cov59	Reverse complement
	LZCU01210302.1_scaffold973465_cov59	None
	LZCU01048762.1_scaffold17933_cov44	Reverse complement
	LZCU01157810.1_scaffold40939_cov53_mid_whole	Gap partially filled with SRA data
	LZCU01012722.1_scaffold1175651_cov61	None
	LZCU01126896.1_scaffold2650035_cov73	None
	LZCU01085103.1_scaffold2534474_cov61	None

Supplementary Table S1: Details of cephalochordate genomic data used in Fig. 1A (mVISTA plot)

	5'	3'	AMPLICON (*B.lan*)	AMPLICON (*A.luc*)
Pax3/7a	CTGGAGGAAGCAGCAGGG	GCCCAGTCCGTTCACCAA	*CTGGAGGAAGCAGCAGGG*CGCCCAGCAGATACC<br>GTCCTTCATGCCGCTGGGATACCCCGCTCCCGCC<br>GCCGGGACACCCACCTACATGCCGGTCCCCGAGC<br>CCGCATACCCAACTAGCGCTCAAGATTCGGCAACC<br>CTGCACAGACCGCAGCCTTTGCCCCCCACCAGCA<br>TCCATCAGAGCGGGGCTGCAGACACGGCCAGCAT<br>GTACGGCACTGCCAGCAAACCTTACCAGTACCCTT<br>TCACCGACTCCTACCTGTCCTCGGGATCCACCGGT<br>CTGTCTGTCAGCAGTGGAATGTCGACTGG*GTTGGT*<br>*GAACGGACTGGGC*	*CTGGAGGAAGCAGCAGGG*CGCCCAGCAGATACC<br>GTCCTTCATGCCGCTGGGATACCCCGCTCCCGCC<br>GCCGGGACACCCACCTACATGCCGGTCCCCGAGC<br>CCGCATACCCAACTAGCGCTCAAGATTCGGCAACC<br>CTGCACAGACCGCAGCCTTTGCCCCCCACCAGCA<br>TCCATCAGAGCGGGGCTGCAGACACGGCCAGCAT<br>GTACGGCACTGCCAGCAAACCTTACCAGTACCCTT<br>TCACCGACTCCTACCTGTCCTCGGGATCCACCGGT<br>CTGTCTGTCAGCAGTGGAATGTCGACTGG*GTTGGT*<br>*GAACGGACTGGGC*
Pax3/7b	CTTCAACCACCTGCTACCCA	CCCGTACTGATAGGTGTCCATG	*CTTCAACCACCTGCTACCCA*TGGGCTACCCCGCC<br>GGAGCCACTGCGCATGCGTCAGCAGCCTACGTCC<br>CGCTGGCGGACACCAGTTATCCCATCACTTCACAA<br>GTAGAATCGACCACCTTGCACCGACCGCAGCCCC<br>TCCCCCCGTCTACGGTGCACCAGAGCATGGCGGC<br>CGACTCCACGTCCTCGTACGGCACGGCCAGCAAG<br>GCGTACCAGTTCACGGGCCTCTCCGACTCCTTCGT<br>CGCCACCGCAGCCAACCACAACACGCTCTCTGGA<br>GGGGTATTGAACATCCTAGGCAACCCCAGCACGT<br>GCCAACAAGACTACCCTCTCGCCTCGCTCTCAACC<br>TCCATGGACTCCACGCAAGCCACCAGCTCAGAAA<br>GCTTCACGGCGCATGCGTCGACCCACGACAGCTA<br>CGCCGCGCATGCTCAGTCGTACTGCACCCCGCCT<br>AGCTACTCGACCACCGCCCACCCGTACAG*CATGG*<br>*ACACCTATCAGTACGGG*	*CTTCAACCACCTGCTACCCA*TGGGCTACCCCGCC<br>GGAGCCACTGCGCATGCGTCAGCAGCCTACGTCC<br>CGCTGGCGGACACCAGTTATCCCATCACTTCACAA<br>GTAGAATCGACCACGTTGCACCGACCACAGCCCC<br>TCCCCCCGTCTACGGTGCACCAGAGCATGGCGGC<br>CGACTCCACGTCCTCGTACGGCACGGCCAGCAAG<br>GCGTACNAGTTCACGGGCCTCTCCGACTCCTTCGT<br>CGCCACCGCAGCCAATCACAACACGCTCTCTGGA<br>GGGGTGTTGAACATCCTCGGCAACCCCAGCACGT<br>GCCAACAAGACTACCCTCTCGCCTCGCTCTCAACC<br>TCCATGGACTCCACGCAAGCCACCAGCTCAGAAA<br>GCTTCACGGCGCATGCGTCGACACACGACAGCTA<br>CGCCGCGCATGCTCAGTCGTACTGCACCCCGCCT<br>AGCTACTCGACCACCGCCCACCCGTACAG*CATGG*<br>*ACACCTATCAGTACGGG*

Supplementary Table S2: Details of primers and amplicons used as probes in WM*IS*H experiment (Figs. 3, 4, S1)

Group/Species	Accession details		
VERTEBRATES	Pax3	Pax7	-
Homo sapiens	NP_852123.1	NP_001128726.1	-
Mus musculus	AAH48699.1	NP_035169.1	-
Gallus gallus	BAB85652.1	XP_015152318.1	-
Python bivitattus	XP_007425723.1	XP_007424290.1	-
Danio rerio (a)	AAC41253.1	CAM12909.1	-
Scyliorhinus torazame	BAM15710.1	BAM15711.1	-
Petromyzon marinus	-	AAL04156.1	-
CEPHALOCHORDATES	Pax3/7a	Pax3/7b	-
Branchiostoma lanceolatum	MF979121	MF979122	(new)
	ACE79721.1	-	(old)
Branchiostoma floridae	MF979123	MF979124	(new)
	EEN66816.1	-	(old)
Branchiostoma belcheri	MF979125	MF979126	(new)
	-	ABK54280.1	(old)
Asymmetron lucayanum	MF979127	MF979128	(new)
	-	-	(old)
OTHER DEUTEROSTOMES	Pax3/7	-	-
Halocynthia roretzi	BAA12289.1	-	-
Saccoglossus kowalevskii	XP_006823827.1	-	-
ARTHROPODS	paired	gooseberry	gooseberry-neuro
Tribolium castaneum	NP_001071090.1	EFA09269.2	EFA09140.1
OTHER PROTOSTOMES	Pax3/7	-	-
Crassostrea gigas	EKC41820.1		
Capitella teleta	ABC68267.1		

Supplementary Table S3: Details of accession numbers used in alignment/phylogenetic analyses

<table>
<thead>
<tr><th>SPECIES</th><th>ACCESSION</th><th>GENE</th><th>ISO-FORM</th><th># EXONS</th><th>EXON LENGTHS, AA</th><th>PRD BOX POS'N</th><th>LEN ^</th><th>HOM. DOM. POS'N</th><th>LEN ^</th><th>Pax7 DOM. POS'N</th><th>LEN ^</th></tr>
</thead>
<tbody>
<tr><td>Mus musculus</td><td>AAH48699.1</td><td>3</td><td>b</td><td>9</td><td>28|78|43|45|68|55|71|82|11*</td><td>34-159</td><td>125</td><td>222-275</td><td>53</td><td>347-391</td><td>44</td></tr>
<tr><td>Homo sapiens</td><td>NP_852124.1</td><td>3</td><td>3</td><td>8</td><td>28|78|43|45|68|55|71|89*</td><td>34-159</td><td>125</td><td>222-275</td><td>53</td><td>347-391</td><td>44</td></tr>
<tr><td>Gallus gallus</td><td>BAB85652.1</td><td>3</td><td>-</td><td>6</td><td>partial chromosome 47|68|55|71|82|11*</td><td>34-161</td><td>195</td><td>222-275</td><td>53</td><td>347-391</td><td>44</td></tr>
<tr><td>Alligator mississippiensis</td><td>XP_006266132.1</td><td>3</td><td>X2</td><td>9</td><td>28|78|43|45|68|55|71|82|11*</td><td>34-159</td><td>193</td><td>222-275</td><td>53</td><td>347-391</td><td>44</td></tr>
<tr><td>Danio rerio</td><td>NP_571352.1/AAC41253.1</td><td>3a</td><td>-</td><td>8</td><td>28|78|43|48|66|55|73|116*</td><td>34-159</td><td>193</td><td>223-276</td><td>53</td><td>350-394</td><td>44</td></tr>
<tr><td>Danio rerio</td><td>NP_001315326.1</td><td>3b</td><td>-</td><td>9</td><td>28|78|42|51|66|55|62|73|11*</td><td>34-158</td><td>192</td><td>225-278</td><td>53</td><td>346-385</td><td>39</td></tr>
<tr><td>Callorhinchus milii</td><td>XP_007887991.1</td><td>3</td><td>-</td><td>13</td><td>56|66|58|19|14|77|43|45|66|55|71|82|11*</td><td>220-346</td><td>126</td><td>405-458</td><td>53</td><td>543-574</td><td>31</td></tr>
<tr><td>Mus musculus</td><td>NP_035169.1</td><td>7</td><td>-</td><td>9</td><td>28|78|43|43|66|55|67|82|38*</td><td>34-159</td><td>193</td><td>218-271</td><td>53</td><td>345-383</td><td>38</td></tr>
<tr><td>Homo sapiens</td><td>NP_002575.1/CAA65522.1</td><td>7</td><td>1</td><td>8</td><td>28|78|43|45|66|55|67|136*</td><td>34-163</td><td>197</td><td>220-273</td><td>53</td><td>347-385</td><td>38</td></tr>
<tr><td>Gallus gallus</td><td>NP_990396.1</td><td>7</td><td>-</td><td>10</td><td>28|78|43|43|66|55|66|22|82|38*</td><td>34-159</td><td>193</td><td>218-271</td><td>53</td><td>352-382</td><td>30</td></tr>
<tr><td>Danio rerio</td><td>XP_009304561.1/CAM12909.1</td><td>7a</td><td>X1</td><td>9</td><td>28|78|43|43|66|55|65|83|38*</td><td>34-161</td><td>195</td><td>218-271</td><td>53</td><td>338-381</td><td>43</td></tr>
<tr><td>Danio rerio</td><td>NP_001139621.1</td><td>7b</td><td>-</td><td>9</td><td>28|78|52|43|65|55|65|83|38*</td><td>34-170</td><td>204</td><td>226-279</td><td>53</td><td>346-389</td><td>43</td></tr>
<tr><td>Callorhinchus milii</td><td>XP_007896063.1</td><td>7</td><td>-</td><td>9</td><td>28|78|43|43|67|55|58|82|38*</td><td>34-161</td><td>195</td><td>219-272</td><td>53</td><td>344-378</td><td>34</td></tr>
<tr><td>A.mississippiensis</td><td>XP_014457309.1</td><td>7</td><td>-</td><td>9</td><td>46|43|43|66|55|66|82|38*</td><td></td><td>0</td><td>157-210</td><td>53</td><td>283-321</td><td>38</td></tr>
<tr><td>Lethenteron japonicum</td><td>KE993866.1:1</td><td>7</td><td>-</td><td>9</td><td>18|79|43|49|69|54|68|90|39*</td><td>24-149</td><td>173</td><td>215-268</td><td>53</td><td>347-380</td><td>33</td></tr>
<tr><td>Saccoglossus kowalevski</td><td>XP_006823827.1</td><td>3B-I</td><td></td><td>6</td><td>16|162|66|55|51|90*</td><td>17-141</td><td>158</td><td>202-254</td><td>52</td><td></td><td></td></tr>
<tr><td>B. lanceolatum</td><td>MF979121</td><td>3/7a</td><td></td><td>7</td><td>23| 162 |65|49|60|79|42*</td><td>24-151</td><td>175</td><td>207-260</td><td>53</td><td>320-364</td><td>44</td></tr>
<tr><td>B. lanceolatum</td><td>MF979122</td><td>3/7b</td><td></td><td>6</td><td>| 178 |62|54|59|79|39*</td><td>9-136</td><td>143</td><td>197-250</td><td>53</td><td>316-357</td><td>41</td></tr>
<tr><td>Ciona intestinalis</td><td>XP_018669403.1</td><td>3/7</td><td></td><td>10</td><td>15|55|49|38|75|75|85|95|96|39*</td><td>16-143</td><td>127</td><td>192-245</td><td>53</td><td></td><td></td></tr>
</tbody>
</table>

Supplementary Table S4: Details of exon/intron structure for a selection of Pax3/7 homologues, showing the conserved exon structure

Supplementary File 2: FASTA format alignment of Bilaterian Pax3/7 genes

```
>Pax4/6__B._floridae_(ROOT)/1-483
------MPHKAWTLQRPADEHAQYS----PVQ-------------------ADPGHSGVNQLGGVFVGGRPLPDSTRRKIVELAHQGARP
CDISRLLQVSNGCVSKILGRYYETGSIRPRAIGGSKPR-VATPEVVAKIAQFKRECPSIFAWEIRDRLLSEGICTNENIPS-----VSSI
NRVLRNLA---SGEKNTLQS-LQSADPQMLEKLRLLNGNAWPHPGPWPYPPSTAGAPPPQTNGNVTTKKEGDGKLASQILTLHGYQDQGD
GSNDDSDEAQARLRLKRKLQRNRTSFTQEQIEALEKEFERTHYPDVFARERLAAKIDLPEARIQVWFSNRRAKWRREEKLRN--QRRSQ-
DSDSSSP-----SRIP---------------------------------ISSS----FSTATMYQ-------------------------
-------------PIAPPSAPVMSR------------------------------------------SSHAGLTDSYSSLPPVPSFS---
------------------------------------------------------------------VPGNMAPMPSMQ------------
-------------------------QSRDQTSYSCMIPHSTAM----------------------------------------TPR---G
YDSLALGSY---NPTHAG-------------------------HHVTTTHPSHMQAPSMPGH--SHMSHANGGSAGL-ISPGVS------
-VPVQVPGAVTEEMT---------------SQPYWPRIQ

>PAX3__H._sapiens/1-484
---MTTLAGAVPRMMRPGPGQNYPR-SGFPLEVSTPL-----------------GQGRVNQLGGVFINGRPLPNHIRHKIVEMAHHGIRP
CVISRQLRVSHGCVSKILCRYQETGSIRPGAIGGSKPKQVTTPDVEKKIEEYKRENPGMFSWEIRDKLLKDAVCDRNTVPS-----VSSI
SRILRSKF---GKGEEEEAD-LERKEA---------------------------------EESEKKAKHSIDGILSERASA-----PQSD
E-G-SDIDSEPDLPLKRKQRRSRTTFTAEQLEELERAFERTHYPDIYTREELAQRAKLTEARVQVWFSNRRARWRKQAGANQ---LMAF-
-NHL-IP-----GGFP--------PTA-------------MPTLPTYQLSETS----YQPTSIPQAVSDPSS------------------
-------------TVHRPQPLPPST-VHQSTI---P------------SNPDSSSAYCLPSTR----HGFSSYTDSFVP-PSGPSNPMNP
TIGNGLSPQ----VMGLLT-----------------------------------------------NHGGVPHQPQTD------------
-------------------------YALSPL--TGGLEPTTTV----------------------------------SASC--SQR----
LDHMKSLDS---LPTSQSYCP------------------P---TYSTTG--YSMDP-V-TGY--QY-GQYGQSAFHY-LKPDIA------
---------------------------------------

>PAX3__M._musculus/1-484
---MTTLAGAVPRMMRPGPGQNYPR-SGFPLEVSTPL-----------------GQGRVNQLGGVFINGRPLPNHIRHKIVEMAHHGIRP
CVISRQLRVSHGCVSKILCRYQETGSIRPGAIGGSKPKQVTTPDVEKKIEEYKRENPGMFSWEIRDKLLKDAVCDRNTVPS-----VSSI
SRILRSKF---GKGEEEEAD-LERKEA---------------------------------EESEKKAKHSIDGILSERASA-----PQSD
E-G-SDIDSEPDLPLKRKQRRSRTTFTAEQLEELERAFERTHYPDIYTREELAQRAKLTEARVQVWFSNRRARWRKQAGANQ---LMAF-
-NHL-IP-----GGFP--------PTA-------------MPTLPTYQLSETS----YQPTSIPQAVSDPSS------------------
-------------TVHRPQPLPPST-VHQSTI---P------------SNADSSSAYCLPSTR----HGFSSYTDSFVP-PSGPSNPMNP
TIGNGLSPQ----VMGLLT-----------------------------------------------NHGGVPHQPQTD------------
-------------------------YALSPL--TGGLEPTTTV----------------------------------SASC--SQR----
LEHMKNVDS---LPTSQPYCP------------------P---TYSTAG--YSMDP-V-TGY--QY-GQYGQSAFHY-LKPDIA------
---------------------------------------

>PAX3__G._gallus/1-484
---MTTLAGAVPRMMRPGAGQSYPR-GGFPLEVSTPL-----------------GQGRVNQLGGVFINGRPLPNHIRHKIVEMAHHGIRP
CVISRQLRVSHGCVSKILCRYQETGSIRPGAIGGSKPKQVTTPDVEKKIEEYKRENAGMFSWEIRDRLLKDGVCDRNTVPS-----VSSI
SRILRSKF---GKGEEEEAE-LERKEA---------------------------------EEGDKKAKHSIDGILSERASA-----AQSD
E-G-SDIDSEPDLPLKRKQRRSRTTFTAEQLEELERAFERTHYPDIYTREELAQRAKLTEARVQVWFSNRRARWRKQAGANQ---LMAF-
-NHL-IP-----GGFP--------PSA-------------MPTLPTYQLSEPS----YQPTSIPQAVSDPSS------------------
-------------TVHRPQPLPPST-VHQSSL---P------------SNPESSSAYCLPSTR----HGFSSYTDSFVP-PSGPSNPMNP
AIGNGLSPQ----VMGLLT-----------------------------------------------NHGGVPHQPQTD------------
-------------------------YALSPL--TGGLEPTTTV----------------------------------SASC--SQR----
LDHMKSLDS---LPTSQSYCP------------------P---TYSTTG--YSMDP-V-TGY--QY-GQYGQSAFHY-LKPDIA------
---------------------------------------

>PAX3__P._bivittatus/1-484
---MTTLGGAVPRMMRPAPGQNYPR-SGFPLEVSTPL-----------------GQGRVNQLGGVFINGRPLPNHIRHKIVEMAHHGIRP
CVISRQLRVSHGCVSKILCRYQETGSIRPGAIGGSKPKQVTTPDVEKKIEEYKRENAGMFSWEIRDKLLKDGVCDRNTVPS-----VSSI
SRILRSKF---GKGEEEEGE-LERKEA---------------------------------EENEKKAKHSIDGILSERASA-----PQSD
E-G-SDIDSEPDLPLKRKQRRSRTTFTAEQLEELERAFERTHYPDIYTREELAQRAKLTEARVQVWFSNRRARWRKQAGANQ---LMAF-
-NHL-IP-----GGFP--------PTA-------------MPTLPTYQLSESS----YQPTSIPQAVSDPSS------------------
-------------TVHRPQPLPPST-VHQSSL---P------------SNPDSSSAYCLPSTR----HGFSSYTDSFVP-PSGPSNPMNP
AIGNGLSPQ----VMGLLT-----------------------------------------------NHGGVPHQPQTD------------
-------------------------YALSPL--TGGLEPTTTV----------------------------------SASC--SQR----
LDHMKSLDS---LPTSQSYCP------------------P---TYSTTG--YSMDP-V-PGY--QY-GQYGQSAFHY-LKPDIA------
---------------------------------------

>Pax3__D._rerio/1-509
---MTALAGGISRMMRPTPPQNYPR-GGYSLEVSTPL-----------------GQGRVNQLGGVFINGRPLPNHIRHKIVEMAHHGIRP
CVISRQLRVSHGCVSKILCRYQETGSIRPGAIGGSKPKQSTTPDVEKKIEEYKRENPGMFSWEIRDKLLKDGICDRNNVPS-----VSSI
SRMLRCKFGGNGDEDEDDDE-VEKREI---------------------------------EENERRAKHSIDGILGDRSS-------HSD
E-G-SDVDSEPGLPLKRKQRRSRTTFTAEQLEELERAFERTHYPDIYTREELAQRAKLTEARVQVWFSNRRARWRKQAGANQ---LMAF-
-NHL-IP-----GGFP--------PSA-------------MSSLQPYQLADSP----YPPSSISQ-VSEQPS------------------
-------------TVHRPQPLPPTS-VHQSGLGSGP------------GAQEGSSAYCLSSGR----HGFSGYSDGYVA-APGHANPVNP
SISNGLPSQ----VMGLL------------------------------------------------NPGAVPHQPQSD------------
-------------------------FALSPL--TGGLEPSTGM----------------------------------PASCHSSQR----
LEALPGLPSMPALPSSQSYCP------------------S---SYSSPG--YSVDH-V-ASY--QY-SQYGQSKVNTSTVTTVERLSRVY
PLKR----------ASFGLVE------------------

>Pax7__S._torazame/1-495
---MTSLTGTAPRMMRPSPGQSYSR-TGFPLEVSTPL-----------------GQGRVNQLGGVFINGRPLPNHIRHKIVEMAHHGIRP
```

CVISRQLRVSHGCVSKILCRYQETGSIRPGAIGGSKPRQVATPDVEKKIEEYKRENPGMFSWEIRDKLLKDGICDRSSVPS-----VSSI
SRVLRAKF---GRRDDDE-E-MEKKD-----------------------------------EDGVRKTKHSIDGILGEKGN-------RLD
D-GASDVESEPDLPLKRKQRRSRTTFTAEQLEELEKAFERTHYPDIYTREELAQRTKLTEARVQVWFSNRRARWRKQAGANQ---LAAF-
-NHL-LP-----GGFP--------PTG-------------MPTLPPYQLPEAS----YPGTTLSQ---DGGS------------------
-------------TVHRPQPLPPSS-MHQSGL----------------TSAAESGSYGLAPNR----HTFSSYPESFMN-ATGANNPMN-
--------Q----VMSILS---NPGAVPPQPQHD------------
-------------------------FSISPL--HGSLDTSNPL----------------------------------SASC--SQR----
SDSIKGVES---LPSSQSYCT------------------P---AYSTSG--YSMDP-V-PGY--QY-AQYGQSAVDY-LTKNVS------
-LSTQRRMKLSEHSAVLGLLQV----ET--GQAY-----

>PAX7__H._sapiens/1-505
---MAALPGTVPRMMRPAPGQNYPR-TGFPLEVSTPL-----------------GQGRVNQLGGVFINGRPLPNHIRHKIVEMAHHGIRP
CVISRQLRVSHGCVSKILCRYQETGSIRPGAIGGSKPRQVATPDVEKKIEEYKRENPGMFSWEIRDRLLKDGHCDRSTVPSG---LVSSI
SRVLRIKF---GKKEEED-E-ADKKE-----------------------------------DDGEKKAKHSIDGILGDKGN-------RLD
E-G-SDVESEPDLPLKRKQRRSRTTFTAEQLEELEKAFERTHYPDIYTREELAQRTKLTEARVQVWFSNRRARWRKQAGANQ---LAAF-
-NHL-LP-----GGFP--------PTG-------------MPTLPPYQLPDST----YPTTTISQ---DGGS------------------
-------------TVHRPQPLPPST-MHQGGLAAAA------------AAADTSSAYG---AR----HSFSSYSDSFMN-PAAPSNHMNP
-VSNGLSPQ----VMSILG---NPSAVPPQPQAD------------
-------------------------FSISPL--HGGLDSATSI----------------------------------SASC--SQR----
ADSIKPGDS---LPTSQAYCP------------------P---TYSTTG--YSVDP-V-AGY--QY-GQYGQTAVDY-LAKNVS------
-LSTQRRMKLGEHSAVLGLLPV----ET--GQAY-----

>PAX7__M._musculus/1-503
---MAALPGAVPRMMRPGPGQNYPR-TGFPLEVSTPL-----------------GQGRVNQLGGVFINGRPLPNHIRHKIVEMAHHGIRP
CVISRQLRVSHGCVSKILCRYQETGSIRPGAIGGSKPRQVATPDVEKKIEEYKRENPGMFSWEIRDRLLKDGHCDRSTVPS-----VSSI
SRVLRIKF---GKKEDDE-E-GDKKE-----------------------------------EDGEKKAKHSIDGILGDKGN-------RLD
E-G-SDVESEPDLPLKRKQRRSRTTFTAEQLEELEKAFERTHYPDIYTREELAQRTKLTEARVQVWFSNRRARWRKQAGANQ---LAAF-
-NHL-LP-----GGFP--------PTG-------------MPTLPPYQLPDST----YPTTTISQ---DGGS------------------
-------------TVHRPQPLPPST-MHQGGLAAAA------------AAADTSSAYG---AR----HSFSSYSDSFMN-PGAPSNHMNP
-VSNGLSPQ----VMSILS---NPSAVPPQPQAD------------
-------------------------FSISPL--HGGLDSASSI----------------------------------SASC--SQR----
ADSIKPGDS---LPTSQSYCP------------------P---TYSTTG--YSVDP-V-AGY--QY-SQYGQTAVDY-LAKNVS------
-LSTQRRMKLGEHSAVLGLLPV----ET--GQAY-----

>PAX7__G._gallus/1-502
---MAALPGTVPRMMRPAPGQNYPR-TGFPLEVSTPL-----------------GQGRVNQLGGVFINGRPLPNHIRHKIVEMAHHGIRP
CVISRQLRVSHGCVSKILCRYQETGSIRPGAIGGSKPRQVATPDVEKKIEEYKRENPGMFSWEIRDRLLKDGHCDRSTVPS-----VSSI
SRVLRIKF---GKKEEEE-D-CDKKE-----------------------------------EDGEKKAKHSIDGILGDKGN-------RLD
E-G-SDVESEPDLPLKRKQRRSRTTFTAEQLEELEKAFERTHYPDIYTREELAQRTKLTEARVQVWFSNRRARWRKQAGANQ---LAAF-
-NHL-LP-----GGFP--------PTG-------------MPTLPPYQLPDST----YPTTTISQ---DGGS------------------
-------------TVHRPQPLPPST-MHQGGL-AAA------------AAADSSSAYG---AR----HSFSSYSDSFMN-AAAPANHMNP
-VSNGLSPQ----VMSILS---NPSGVPPQPQAD------------
-------------------------FSISPL--HGGLDTTNSI----------------------------------SASC--SQR----
SDSIKSVDS---LPTSQSYCP------------------P---TYSTTS--YSVDP-V-AGY--QY-GQYGQTAVDY-LTKNVS------
-LSTQRRMKLGEHSAVLGLLPV----ET--GQAY-----

>PAX7__P._bivittatus/1-501
---MAALPGTVPRMMRPAPGQNYPR-TGFPLEVSTPL-----------------GQGRVNQLGGVFINGRPLPNHIRHKIVEMAHHGIRP
CVISRQLRVSHGCVSKILCRYQETGSIRPGAIGGSKPRQVATPDVEKKIEEYKRENPGMFSWEIRDRLLKDGHCDRTTVPS-----VSSI
SRVLRIKF---GKKEEED-D-CEKKE-----------------------------------EDCEKKAKHSIDGILGDKGS-------RLD
E-G-SDVESEPDLPLKRKQRRSRTTFTAEQLEELEKAFERTHYPDIYTREELAQRTKLTEARVQVWFSNRRARWRKQAGANQ---LAAF-
-NHL-LP-----GGFP--------PAG-------------MPTLPPYQLPDST----YPTTTLSQ---DGGS------------------
-------------TVHRPQPLPPST-MHQGGL--SA------------TTGDSNSAYG---AR----HGFSSYTDSFMN-PTAPANHMNP
-VSNGLSPQ----VMSILS---HPSGAPPPAPAD------------
-------------------------FSISPL--HGGLESTNSL----------------------------------SASC--SQR----
SESIKSVDS---LPTSQAYCP------------------P---TYTSTG--YSVDP-V-AGY--QY-GQYSQTAVDY-LTKNVS------
-LSTQRRMKLGEHSAVLGLLPV----ET--GQAY-----

>Pax7__D._rerio/1-507
---MATLPGTVPRMMRPAPGQNYPR-TGFPLEVSTPL-----------------GQGRVNQLGGVFINGRPLPNHIRHKIVEMAHHGIRP
CVISRQLRVSHGCVSKILCRYQETGSIRPGAIGGSKPRQVATPDVEKRIEEYKRENPGMFSWEIRDKLLKDGVCDRGTVPSGEASSVSSI
SRVLRARF---GKKDDDD-E-CDKKD-----------------------------------EDGEKKTKHSIDGILGDKGN-------RTD
E-G-SDVESEPDLPLKRKQRRSRTTFTAEQLEELEKAFERTHYPDIYTREELAQRTKLTEARVQVWFSNRRARWRKQAGANQ---LAAF-
-NHL-LP-----GGFP--------PTG-------------MPTLPTYQLPEST----YPSTTLSQ---DGSS------------------
-------------TLHRPQPLPPSS-MHQGGL-----------------SADSGSAYGLSSNR----HTFSSYSETFMS-PTASSNHMNP
-VSNGLSPQ----VMSILS---NPSAVPSQPQHD------------
-------------------------FSISPL--HGGLEASNPI----------------------------------SASC--SQR----
SDPIKSVDS---LASTQSYCP------------------P---TYSATS--YSVDP-VTAGY--QY-SQYGQTAVDY-LAKNVS------
-LSTQRRMKLGDHSAVLGLLQV----ET--GQAY-----

>Pax3__S._torazame/1-506
---MSTLAGPLPRMMRPAPGQSYPRSSGFPLEVSTPL-----------------GQGRVNQLGGVFINGRPLPNHIRHKIVEMAHHGIRP
CVISRQLRVSHGCVSKILCRYQETGSIRPGAT-GSKPRQMAIPDVEKKIEEYKKENPGMFSWEIRDKLLKDGMCDRNTIPS-----VSSI
SRILRSRF---GKKEDEEDD-CERKEY----------------------------------EEGEKKTKHSIDGILANKAN-------NSD
E-A-SDIDSEPDLPLKRKQRRSRTTFTAEQLEELERAFERTHYPDIYTREELAQRAKLTEARVQVWFSNRRARWRKQAGANQ---LLAF-
-NHL-IP-----GGFP--------PTA-------------MPALSPYQLSDAS----YPPTSIPQAISDPSN------------------
-------------TVHRPQPLPPTS-VHQSSL---P------------STPDSSSAYCLSTSR----HGFSGYTDSFVS-PTGSTNPMNP

```
TVSNGLSPQ----VMGLLA-----------------------------------------------NPGGVPHQPQTD------------
-------------------------YALSPL--TGGLEPSTSV----------------------------------SASC--SQR----
LDPMKPLDS---LPTTQSYCP------------------S---TYSSSG--YSMDS-V-AGY--QY-GQYGQSKCHVNLSNTFSYKNAYP
PHPSSICIKMCDSVYKL----------------------

>Pax7__P._marinus/1-505
-------------MMRPAPGQNYPR-SGFPLEVSTPL-----------------GQGRVNQLGGVFINGRPLPNHIRHKIVELAHHGVRP
CVISRQLRVSHGCVSKILCRYQETGSIRPGAIGGSKPRQVASTDVERKIEEYKRENPGMFSWEIRDRLLKDGVCDRASVPS-----VSSI
SRILRAKF---GKRDEEEEEGAEKKDF--------------------------------GDDGDKKAKHSIDGILGDKAG-------STV
E-DSSDVDSEPDLPLKRKQRRSRTTFTAEQLEELEKAFERTHYPDIYTREELAQRTKLTEARVQVWFSNRRARWRKQAGANQ---LAAF-
-NHL-IP-----GGFP--------PAS-------------MPGLPPYQLPEGP----YPGSGIPQ--VDTGT------------------
-------------TVHRPQPLPPSS-MHQGGG---L------------AGGDGAGAYGLSSNR----SGF--YSDSFMSAASAPSNHMAP
-MANGLSPQ----MMGILG-----------------------------------------------NPSAMPPQHGQE------------
-------------------------YSLSPLPSSGLVDHSSGL----------------------------------SASC--SQRGGGG
GDGMKHGDG---LPPP-GLLP------------------A---TYAAAG--YGLEP-MPAAY--QY-GQYGQTAADY-LAKNVG------
-LGGQRRLKLGEHSAVLGLLQV---AET--GQAY-----

>Pax3/7b__A._lucayanum/1-469
--------------------MALF----FVLP----------------------GQGRVNQLGGVFINGRPLPNHIRHKIVEMAAAGIRP
CVISRQLRVSHGCVSKXXXXXXXXXXXXXXXXGGSKPR-VATPEVEKKIEDYKRDNPGMFSWEIRDHLLKDGMCDRSTVPS-----VSSI
SRILRGGKGDGRRSDD-DSE-DERKDG---------------------------------EDGEKKRSHSIDGILGEKGG-------CRG
E-D-SDCDSEPDLPLKRKQRRSRTTFTPEQLDELEKAFERTHYPDIYTREELAQRTKLTEARVQVWFSNRRARWRKQAGAQQ---LAGF-
-NHL-LP-----MGYPAGATAHASAAY-------------VP------LADTS----YPITSQVE-----ST------------------
-------------TLHRPQPLPPST-VHQSMA------------------ADSTSSYGTAGKA----YQFTGLSDSFVATAAN-------
--HNTLSGG----VLNILG-----------------------------------------------NPST---CQ-QE------------
-------------------------YPLASLSTSMDSTQATSS---------------------------------ESFTAHAS------
-----SHDS-YAAH-AQSYCT------------------PP--SYSTTAHPYSMDS-YQYGS--QYPSQ-GXXXXXX-XXXXXX------
--XXXXXXXXXXX--XXXXXXX---XXXX-XXXX-----

>Pax3/7b__B._belcheri/1-470
--------------------MALF----FVLP----------------------GQGRVNQLGGVFINGRPLPNHIRHKIVEMAAAGIRP
CVISRQLRVSHGCVSKILCRYQETGSIKPGAIGGSKPR-VATPEVEKKIEDYKRDNPGMFSWEIRDRLLKDGMCDRSTVPS-----VSSI
SRILRGGKGDGRRSEDGDSE-DERRDG---------------------------------EDGEKKRSHSIDGILGEKGG-------CRG
E-D-SDCDSEPDLPLKRKQRRSRTTFTPEQLEELEKAFERTHYPDIYTREELAQRTKLTEARVQVWFSNRRARWRKQAGAQQ---LAGF-
-NHL-LP-----MGYPAGATAHASAAY-------------VP------LADTS----YPITSQVE-----ST------------------
-------------TLHRPQPLPPST-VHQSMA------------------ADSTSSYGTAGKA----YQFTGLSDSFVATAAN-------
--HNTLSGG----VLNILG-----------------------------------------------NPST---CQ-QD------------
-------------------------YPLASLSTSMDSTQATSS---------------------------------ESFTAHAS------
-----THDS-YAAH-AQSYCT------------------PP--SYSTTAHPYSMDS-YQYGS--QYPSQ-GSCTSSY-GSTGTS------
--LSSLRQKSLVH--PLGMIPV---GSGT-GQAY-----

>Pax3/7b__B._floridae/1-468
--------------------MALF----FVLP----------------------GQGRVNQLGGVFINGRPLPNHIRHKIVEMAAAGIRP
CVISRQLRVSHGCVSKILCRYQETGSIKPGAIGGSKPR-VATPEVEKKIEDYKRDNPGMFSWEIRDRLLKDGMCDRSTVPS-----VSSI
SRILRGGKGDGRRSEDGDSE-DERRDG---------------------------------EDGEKKRSHSIDGILGEKGG-------CRG
E-D-SDCDSEPDLPLKRKQRRSRTTFTPEQLEELEKAFERTHYPDIYTREELAQRTKLTEARVQVWFSNRRARWRKQAGAQQ---LAGF-
-NHL-LP-----MGYPAGATAHASAAY-------------VP------LADTS----YPITSQVE-----ST------------------
-------------TLHRPQPLPPST-VHQSMA------------------ADSTSSYGTAGKA----YQFTGLSDSFVATAAN-------
--HNTLSGG----VLNILG-----------------------------------------------NPST---CQ-QD------------
-------------------------YPLASLSTSMDSTQATSS---------------------------------ESFTAHAS------
-----THDS-YA---AQSYCT------------------PP--SYSTTAHPYSMDT-YQYGS--QYPSQ-GSCTSSY-GSTGTS------
--LSSLRQKSLVH--PLGMIPV---GSGT-GQAY-----

>Pax3/7b__B._lanceolatum/1-470
--------------------MALF----FVLP----------------------GQGRVNQLGGVFINGRPLPNHIRHKIVEMAAAGIRP
CVISRQLRVSHGCVSKILCRYQETGSIKPGAIGGSKPR-VATPEVEKKIEDYKRDNPGMFSWEIRDRLLKDGMCDRSTVPS-----VSSI
SRILRGGKGEGRASEDGDSE-DERRDG---------------------------------EDGEKKRSHSIDGILGEKGG-------CRG
E-D-SDCDSEPDLPLKRKQRRSRTTFTPEQLDELEKAFERTHYPDIYTREELAQRTKLTEARVQVWFSNRRARWRKQAGAQQ---LAGF-
-NHL-LP-----MGYPAGATAHASAAY-------------VP------LADTS----YPITSQVE-----ST------------------
-------------TLHRPQPLPPST-VHQSMA------------------ADSTSSYGTASKA----YQFTGLSDSFVATAAN-------
--HNTLSGG----VLNILG-----------------------------------------------NPST---CQ-QD------------
-------------------------YPLASLSTSMDSTQATSS---------------------------------ESFTAHAS------
-----THDS-YAAH-AQSYCT------------------PP--SYSTTAHPYSMDT-YQYGS--QYPSQ-GSCTSSY-GSTGTS------
--LSSLRQKSLVH--PLGMIPV---GSGT-GQAY-----

>Pax3/7a__A._lucayanum/1-478
--------MNMMTMRQPGGPVATFR-PAFPLE----------------------XXGRVNQLGGVFINGRPLPNHIRHKIVEMAAAGIRP
CVISRQLRVSHGCVSKILCRYQETGSIKPGAIGGSKPR-VATPEVEKKIEDYKRDNPGMFSWEIRDHLLKDGMCDRSTVPS-----VSSI
SRILR---GKGHKISDLELE-A-DEAS---------------------------------SDGDRK-PHSIDGILGEKNG--------LG
D-S-SDCDSEPDXXXXXXXXXSRTTFTPEQLDELEKAFERTHYPDIYTREELAQRTKLTEARVQVWFSNRRARWRKQQGAQQ---MASF-
-----MP-----LGYP-GAPAAGTPTY-------------MP------VPEPA----YPTTTCQD-----SA------------------
-------------TLHRPQPLPPTS-IHQSGA------------------ADTASMYGTASKP----YQYP-FSDTYLSASSAGLS----
-VSNGMSSG----LMNGLG-----------------------------------------------SVSA---QQ-EA------------
-------------------------FPVHCVPAGSN-SPATSI---------------------------------PSTDCYSS-T----
-----AADT-YTFNTAQTYCS------------------TPAHSYSSTAHAYSMD-----GS--W--VQ-GTSSTDF-NSNSLG------
--IAALRQKSREHSAALGLIQV---AGGAMAQAY-----
```

```
>Pax3/7a__B._belcheri/1-481
--------MNNMTMRQAGGPVATFR-PAFPLE----------------------GQGRVNQLGGVFINGRPLPNHIRHKIVEMAAAGIRP
CVISRQLRVSHGCVSKILCRYQETGSIKPGAIGGSKPR-VATPEVEKKIEDYKRDNPGMFSWEIRDRLLKDGMCDRSTVPS-----VSSI
SRILR---GKGHKISDLECE-GDDDTS---------------------------------IDSDRK-PHSIDGILGEKNG--------PG
D-S-SDCDSEPDLPLKRKQRRSRTTFTPEQLEELEKAFERTHYPDIYTREELAQRTKLTEARVQVWFSNRRARWRKQQGAQQ---IPSF-
-----MP-----LGYP--APAAGTPTY-------------MP------VPEPA----YPTNA-PD-----SA------------------
-------------TLHRPQPLPPTS-IHQSGA------------------ADTASMYGTASKP----YQYP-FSDSYLSTASTGLS----
-VSSGMSTG----LVNGLG-----------------------------------------------SMSS---QQVDP------------
-------------------------YPVHCAPASSSTSPATSL---------------------------------PSSDCYSSTT----
-----AADT-YSYNTAQTYCT------------------APAHSFSSTAHAYSMD-----GS--W--VQGGTNSTDF-NSNSLG------
--IAALRQKSREHSAALGLIQV---AGGAMAQAY-----

>Pax3/7a__B._floridae/1-479
--------MNNMTMRQAGGPVATFR-PAFPLE----------------------GQGRVNQLGGVFINGRPLPNHIRHKIVEMAAAGIRP
CVISRQLRVSHGCVSKILCRYQETGSIKPGAIGGSKPR-VATPEVEKKIEDYKRDNPGMFSWEIRDRLLKDGMCDRSTVPS-----VSSI
SRILR---GKGHKISDLEIE-GEDDFS---------------------------------IDGDRK-PHSIDGILGEKNG--------PG
D-S-SDCDSEPDLPLKRKQRRSRTTFTPEQLEELEKAFERTHYPDIYTREELAQRTKLTEARVQVWFSNRRARWRKQQGAQQ---IPSF-
-----MP-----LGYP--APAAGTPTY-------------MP------VPEPA----YPANA-QD-----SA------------------
-------------TLHRPQPLPPTS-IHQNGA------------------ADTASMYGTASKP----YQYP-FSDSYLSSGSTGLS----
-VSSGMSTG----LVGGLG-----------------------------------------------SMGS---QQVEP------------
-------------------------FPVHCAPVSSSSSPATSI---------------------------------PSTDCYSS-T----
-----AADT-YSYNTAQTYCT------------------APAHSYSSSAHAYSMD-----GS--W--VQ-GANSTDF-NSNSLG------
--IAALRQKSREHSAALGLIQV---AGGAMAQAY-----

>Pax3/7a__B._lanceolatum/1-479
--------MNNMTMRQAGGPVATFR-PAFPLE----------------------GQGRVNQLGGVFINGRPLPNHIRHKIVEMAAAGIRP
CVISRQLRVSHGCVSKILCRYQETGSIKPGAIGGSKPR-VATPEVEKKIEDYKRDNPGMFSWEIRDRLLKDGMCDRSTVPS-----VSSI
SRILR---GKGHKISDLEME-GEDELS---------------------------------IDGDRK-PHSIDGILGEKNG--------PG
D-S-SDCDSEPDLPLKRKQRRSRTTFTPEQLDELEKAFERTHYPDIYTREELAQRTKLTEARVQVWFSNRRARWRKQQGAQQ---IPSF-
-----MP-----LGYP--APAAGTPTY-------------MP------VPEPA----YPTNA-QD-----SA------------------
-------------TLHRPQPLPPTS-IHQSGA------------------ADTASMYGTASKP----YQYP-FTDSYLSSGSTGLS----
-VSSGMSTG----LVNGLG-----------------------------------------------SMGS---QQVDP------------
-------------------------YPVHCAPASSSTSPATSI---------------------------------PSTDCYSS-T----
-----AADT-YSYNTAQTYCT------------------APAHSYSSSAHAYSMD-----GS--W--VQ-GANSTDF-NSNSLG------
--IAALRQKSREHSAALGLIQV---AGGAMAQAY-----

>Pax3/7__H._roretzi/1-704
-------------MMHRP---GTFR-PAFPLE----------------------GQGRVNQLGGVFINGRPLPNHIRHKIVEMAAHGIRP
CVISRQLRVSHGCVSKILCRYQETGSIKPGAIGGSKPK-VTNSEIESKIEQYKKDSPSMFSWEIRDQLIKEGLCDRSSAPT-----VSAI
SRILR------SKGCDTSNE-SAEDP----------------------------------ENGNTNSDTSSNGATGER---------EAD
E-GDSDCDSEPELPLKRKQRRSRTTFSADQLEELERCFERTHYPDIYTREELAQRTRLTEARVQVWFSNRRARWRKQMAAQQ---LPAIH
PHHT-HPHLPHHLGAYHQTIAAAGMSAHNYMLQAAAAAQHQPPPSSNPLAQNG----HSSHYAAHHPSAHGHLPTTPTAASAGTSVCGPI
ESSFASPTAHDLGSVHRHTHHPLSSPLHHAHPYSQAGMRYDGSHDAALNVAAVAAGYGLVGAS----AAAARYSAQFPS-TAFGDGY---
HQHHSLSPE--SSIFGVNGSVHSHAPGAADLTVSDYGPRQTESSHVRQHQSSAAVGGSTTRTSENHSPSSSSPRQQHQTQGGISHDSVSG
NGRRSESSAGSHDNHPRNGVSNSTSSPVTTSESHVSGMPES-VYNQRPSNAPMHLQQQSHQHSAAHLQHLRGGPSVDHTSCNGNQMIQSS
QYSLMHRSNVPEHSSIQSAAAYSAGFGVTAD--------P---SMATAAHQYHTTHNPFASC--QY-SGYGQAASDY-ENAGIT------
----ALRMKSREHSAALGLISVGAGGATSMQPAY-----

>Pax3/7__C._intestinalis/1-625
-------------MMHPG---SNFR-PAFPLE----------------------GQGRVNQLGGMFINGRPLPSHVRHKIVEMAAHGVRP
CVISRQLRVSHGCVSKILCRYQETGSIKPGAIGGSKPK-VATADVDNKIEEYKKENPGIFSWEIRERLIKEGICDRSNVPS-----VSSI
SRTLR------AKGCDVENE-SESSA----------------------------------RLDPGNRSSSSGG--------------EPN
EVGGSDSESEPDLPLKRKQRRSRTTFSAEQLDELERCFERTHYPDIYTREELAQRTRLTEARVQVWFSNRRARWRKQMAAQQFHGIPAHH
PHHL-SP----HLGYPHTMPGSVGQAAHNYMLQTSGAHH----------VQSM----HESF--AHTASSHDH----------GS------
-------------SLHRPHASVLTSPVHHA---SQA-IRYDTTD--PLNQSA-AAAYGLMGASAAAAAAAARYS-QYPSAAAFGDAYPIH
YHHHSTSPPSGSSIFGFPSHTRQTATNPVELT--------TGNPHSGPNSSPIDVG----------SPGEAGNHPLHQ-----QHFVLAQ
SARRAHQASNQRNHHNQHHSGETTTAPSTSPASNGDGHPTTGIESQ--SRAYSALAGQQHHNTDLH----------SSTSVAGQQSTAML
SSSVDHSSNVFAGQE-RGAPSSRAGYGVGPSGFATGEVLP---TSSAASHQ------SYASC--QY-SPYGQASGDY-GSAGIA------
----ALRMKSREHSASIGLIPV--GGGPSVQHAY-----

>Pax3/7__S._kowalevskii/1-438
-------------MIQPG---RSFAAPGFPLE----------------------GQGRMNQLGGLFINGRPLPNHIRYKIVEMAHQGIRP
CQISRTLRVSHGCVSKILCRYQETGSIRPGTIGGSKPK-VATVEVEKRIEDYRKDNEGIFSWEIRERLLNEKICDQQNVPS-----ISSI
SRILRHGN---GRHKTNEHEKVCRADG---------------------------------NAERAKNSHSIEGILN-----------KRD
EEPDYEPELQPDLPLKHKQRRCRTTFTCEQLEQLEKAFDRTHYPDIYTREELAQRTGLTEARVQVWFSNRRARWRKQASFLP---LNGY-
-----SP------WYPMATTGSVMPVSAGPPCVLPDTMHSFPSQDFSFLPPSHPSRLYQNSFYTQYNPPTFSLPSMP-------------
-------------DLSTGSKSVVSGPVHQ--------------------------------------HVYQGQPEYVQSSLCKNPIVMNR
PIIPPVSKSPVCSLPMIVN-----------------------------------------------------------------------
-------------------------HPMTPTVTNTPCQPFPSP-----------------------------------------------
---------------------------------------PWVHTMPAGGQEPY-----------QQVSQFSPKHGLYSSFM---------
---------------------------------------

>Gsb__T._castaneum/1-399
--------------MAPYF-------TGYPFQ----------------------GQGRVNQLGGVFINGRPLPNHIRLKIVEMAAAGIRP
CVISRQLRVSHGCVSKILNRYQETGSIRPGVIGGSKPR-VATLEVEARIEQLKKEEPQIFSWEIRDRLIKEGICDKNSAPS-----VSSI
SRLLRG-----G--RKE--------------------------------------------DPD-RKNHSIEGILGPNSS-------CD-
---ESDTESEPGIPLKRKQRRSRTTFTGEQLEALERAFGRTQYPDVYTREELAQKTKLTEARVQVWFSNRRARLRKQLNSQQ---LNAF-
```

```
-NSMSLP-----SAFP------------------------V----QHQYGDPA----FNQSTWAQQSYASAALSSGALPHSALTTGGAST
-------------SVASSTPNTL----YNSY-----------------------NSAATLDQNNQFGYNMAQVSPNQHSVSP-----AAH
QQSAVWSRQNNKVAENL----------------------------------------------------SSWHENYSL------------
------------------------------------------------------------------------------------------
---------------------------------------FAGSHYGAHSPSEAKSGYPYLGTMDMCASRVH-------------------
---------------------------------------

>Gsb-n__T._castaneum/1-338
------------MDMRPIF-------GGYPFQ----------------------GQGRVNQLGGVFINGRPLPNHIRLKIVEMAAAGVRP
CVISRQLRVSHGCVSKILNRYQETGSIRPGVIGGSKPR-VATPEIETRIEQMKKENPTIFSWEIRERLIKEGVTD---PPS-----VSSI
SRLLRG-----GGRRDG--------------------------------------------DPDGKKDYTIDGILGGRE---------E-
---ESDTESEPGIPLKRKQRRSRTTFSGEQLEALERAFSRTQYPDVYTREELAQQTGLTEARIQVWFSNRRARLRKHVGNSN---ALAF-
-PLANMP-----CQYP------------------------AESAHQHDWRNAQ----FTNYNMFQQ-----------------------T
-------------SYTNPQENRD----Y----------------------------SAIFDAN--YATLMAQ------------------
-------KNKESDAKNLGG-----------------------------------------------NPGTSW-ANFQD------------
------------------------------------------------------------------------------------------
---------------------------------------FSGDQY---APKNYWA-----------------------------------
---------------------------------------

>Prd__T._castaneum/1-414
-MTVTGNLGIM----HRHC-------FGYPFQGTTYKYTGCSDNDGFLP-----GQGRVNQLGGVFINGRPLPNHIRLKIVEMAANGIRP
CVISRQLRVSHGCVSKILNRYQETGSIRPGVIGGSKPR-VATPEVENRIEQYKRENPSIFSWEIRDRLVKEGICDRSTAPS-----VSAI
SRLLRGK----GGECDEITIQAILEFS--------------------------------VTN--PTIKEDFCAVLWKDKS-------TDN
EG-VSDCDSEPGIPLKRKQRRSRTTFTAHQLDELEKAFERTQYPDIYTREELAQRTKLTEARIQVWFSNRRARLRKQITSAA---TPLV-
-RSY-AP-----ERYP------------------------P----------------------LAQGVNDDAF----------------N
-------------SVATPTPTSVMTELYPSPV-------------------PGHSTSPNL----PLTHNIAH---------------NPY
PSHSIYPSSNLMPLNNI-Q-----------------------------------------------NLSQS-GINYKD------------
------------------------------------------------------------------------------------------
---------------------------------------TNDNNVSAV----TTQH---QGYPTNTVTILGPNSGNT-PKI---------
------------------LLQI-----------------

>Pax3/7__C._gigas/1-466
MFGFVNGFGVYLINEERLI-------VRYPTAMFSYHLAAL----GLMPSFQMEGQGRVNQLGGVFINGRPLPNHIRLKIVELAAQGVRP
CVISRQLRVSHGCVSKILQRYQETGSIRPGVIGGSKPR-VATPEVEKKIEQYKRENPGIFSWEIRDRLLKEGICDRSSVPS-----VSSI
SRVLRSKFQNIGSDSEDEEPRPSDDIS--------------------------------VNDNEKSSKYSIDGLLADDKS-------EKS
EDEESDCDSEPGLSVKRKQRRSRTTFTAEQLEELERAFERTHYPDIYTREELAQRTKLTEARVQVWFSNRRARWRKQMGSNQ---ISAL-
-NSI-L----------------------------------H----------------------LPQGGASSYL----------------L
-------------HETTPS--------YPLP-----------------------SVSDTLWHRNSMTHHLQNFSSLKTNDSAYAGLMENY
LSHA-------SQMHNL-T-----------------------------------------------NPTDSLASNWCS------------
------------------------------------------------------------------------------------------
---------------------------------------PVTSSVSALAYQTTTNHFNPHGYGDVTKSTLHPYNSHI-ASVSSAERCAID
ESLVALRMKSREHSAALSLMQVTDNKMSASF--------

>Pax3/7__C._teleta/1-416
-------------MLQRL----------FPLG-----------LPPLLPTYGLDGQGRVNQLGGVFINGRPLPNHIRLKIIEMASQGVRP
CVISRTLRVSHGCVSKILQRYQETGSIRPGSIGGSKPR-VATPDVEDRIHDLKKENPGIFSWEIRDRLLKDGVCDRSSVPS-----VSSI
SRVLRSHL---REGDEDKIDDEDEEES--------------------------------KDD-------------------------DDD
DDDSESLTSEPGLSLSRKQRRSRTTFSADQLEHLEKAFDRTHYPDIYTREELAQRSGLTEARVQVWFSNRRARWRKQMGSGQ---LASSF
NSLLSAA-----SGYS---------------------------------STPS----YTPSLNQDHP-----------------------
-------------ALPHPTPSVPAPPIKAESL-------------------------------------PPAFSDPFRQHSHLPYPF---
------------------------------------------------------------------LPGPTPHHPVSQ------------
------------------------------------------------------------------------------------------
----------------------------------------SLMTSVASSWNSAMSSLRSSACLPPYPSPYPPSSMQHPFHYHTSFNAGHE
AGSYPYPSGKVHAPTPIGLGSFGAMAPPSHIVSGL----
```

Supplementary File 3: FASTA format alignment of cephalochordate Pax3/7 genes

```
>Pax3/7a__B._lanceolatum/1-479
MNMMTMRQAGGPVATFRPAFPLEGQGRVNQLGGVFINGRPLPNHIRHKIVEMAAAGIRPCVISRQLRVSHGCVSKILCRYQETGSIKPGAIGG
SKPRVATPEVEKKIEDYKRDNPGMFSWEIRDRLLKDGMCDRSTVPSVSSISRILR---GKGHKISDLEMEGEDELSIDGDRK-PHSIDGILGE
KNG-PGDSSDCDSEPDLPLKRKQRRSRTTFTPEQLDELEKAFERTHYPDIYTREELAQRTKLTEARVQVWFSNRRARWRKQQGAQQIPSF---
MPLGYP--APAAGTPTYMPVPEPAYPTNA-QDSATLHRPQPLPPTSIHQSGAADTASMYGTASKPYQYP-FTDSYLSSGSTGLSVSSGMSTGL
VNGLGSMGSQQVDPYPVHCAPASSSTSPATSIPSTDCYSS-TAADTYSYNTAQTYCTAPAHSYSSSAHAYSMD----GSW--VQ-GANSTDFN
SNSLGIAALRQKSREHSAALGLIQVAGGAMAQAY

>Pax3/7a__B._floridae/1-479
MNMMTMRQAGGPVATFRPAFPLEGQGRVNQLGGVFINGRPLPNHIRHKIVEMAAAGIRPCVISRQLRVSHGCVSKILCRYQETGSIKPGAIGG
SKPRVATPEVEKKIEDYKRDNPGMFSWEIRDRLLKDGMCDRSTVPSVSSISRILR---GKGHKISDLEIEGEDDFSIDGDRK-PHSIDGILGE
KNG-PGDSSDCDSEPDLPLKRKQRRSRTTFTPEQLEELEKAFERTHYPDIYTREELAQRTKLTEARVQVWFSNRRARWRKQQGAQQIPSF---
MPLGYP--APAAGTPTYMPVPEPAYPANA-QDSATLHRPQPLPPTSIHQNGAADTASMYGTASKPYQYP-FSDSYLSSGSTGLSVSSGMSTGL
VGGLGSMGSQQVEPFPVHCAPVSSSSSPATSIPSTDCYSS-TAADTYSYNTAQTYCTAPAHSYSSSAHAYSMD----GSW--VQ-GANSTDFN
SNSLGIAALRQKSREHSAALGLIQVAGGAMAQAY

>Pax3/7a__B._belcheri/1-481
MNMMTMRQAGGPVATFRPAFPLEGQGRVNQLGGVFINGRPLPNHIRHKIVEMAAAGIRPCVISRQLRVSHGCVSKILCRYQETGSIKPGAIGG
SKPRVATPEVEKKIEDYKRDNPGMFSWEIRDRLLKDGMCDRSTVPSVSSISRILR---GKGHKISDLECEGDDDTSIDSDRK-PHSIDGILGE
KNG-PGDSSDCDSEPDLPLKRKQRRSRTTFTPEQLEELEKAFERTHYPDIYTREELAQRTKLTEARVQVWFSNRRARWRKQQGAQQIPSF---
MPLGYP--APAAGTPTYMPVPEPAYPTNA-PDSATLHRPQPLPPTSIHQSGAADTASMYGTASKPYQYP-FSDSYLSTASTGLSVSSGMSTGL
VNGLGSMSSQQVDPYPVHCAPASSSTSPATSLPSSDCYSSTTAADTYSYNTAQTYCTAPAHSFSSTAHAYSMD----GSW--VQGGTNSTDFN
SNSLGIAALRQKSREHSAALGLIQVAGGAMAQAY

>Pax3/7a__A._lucayanum/1-478
MNMMTMRQPGGPVATFRPAFPLEXXGRVNQLGGVFINGRPLPNHIRHKIVEMAAAGIRPCVISRQLRVSHGCVSKILCRYQETGSIKPGAIGG
SKPRVATPEVEKKIEDYKRDNPGMFSWEIRDHLLKDGMCDRSTVPSVSSISRILR---GKGHKISDLELEA-DEASSDGDRK-PHSIDGILGE
KNG-LGDSSDCDSEPDXXXXXXXXXSRTTFTPEQLDELEKAFERTHYPDIYTREELAQRTKLTEARVQVWFSNRRARWRKQQGAQQMASF---
MPLGYP-GAPAAGTPTYMPVPEPAYPTTTCQDSATLHRPQPLPPTSIHQSGAADTASMYGTASKPYQYP-FSDTYLSASSAGLSVSNGMSSGL
MNGLGSVSAQQ-EAFPVHCVPAGSN-SPATSIPSTDCYSS-TAADTYTFNTAQTYCSTPAHSYSSTAHAYSMD----GSW--VQ-GTSSTDFN
SNSLGIAALRQKSREHSAALGLIQVAGGAMAQAY

>Pax3/7b__B._lanceolatum/1-470
------------MALF---FVLPGQGRVNQLGGVFINGRPLPNHIRHKIVEMAAAGIRPCVISRQLRVSHGCVSKILCRYQETGSIKPGAIGG
SKPRVATPEVEKKIEDYKRDNPGMFSWEIRDRLLKDGMCDRSTVPSVSSISRILRGGKGEGRASEDGDSEDERRDGEDGEKKRSHSIDGILGE
KGGCRGEDSDCDSEPDLPLKRKQRRSRTTFTPEQLDELEKAFERTHYPDIYTREELAQRTKLTEARVQVWFSNRRARWRKQAGAQQLAGFNHL
LPMGYPAGATAHASAAYVPLADTSYPITSQVESTTLHRPQPLPPSTVHQSMAADSTSSYGTASKAYQFTGLSDSFVATAAN----HNTLSGGV
LNILGNPSTCQ-QDYPLASLSTSMDSTQATSSESFTAHAS--THDSYAAH-AQSYCTPP--SYSTTAHPYSMDTYQYGSQYPSQ-GSCTSSYG
STGTSLSSLRQKSLVH--PLGMIPVGSGT-GQAY

>Pax3/7b__B._floridae/1-468
------------MALF---
FVLPGQGRVNQLGGVFINGRPLPNHIRHKIVEMAAAGIRPCVISRQLRVSHGCVSKILCRYQETGSIKPGAIGGSKPRVATPEVEKKIEDYKR
DNPGMFSWEIRDRLLKDGMCDRSTVPSVSSISRILRGGKGDGRRSEDGDSEDERRDGEDGEKKRSHSIDGILGEKGGCRGEDSDCDSEPDLPL
KRKQRRSRTTFTPEQLEELEKAFERTHYPDIYTREELAQRTKLTEARVQVWFSNRRARWRKQAGAQQLAGFNHLLPMGYPAGATAHASAAYVP
LADTSYPITSQVESTTLHRPQPLPPSTVHQSMAADSTSSYGTAGKAYQFTGLSDSFVATAAN----HNTLSGGVLNILGNPSTCQ-QDYPLAS
LSTSMDSTQATSSESFTAHAS--THDSYA---AQSYCTPP--SYSTTAHPYSMDTYQYGSQYPSQ-GSCTSSYGSTGTSLSSLRQKSLVH--
PLGMIPVGSGT-GQAY

>Pax3/7b__B._belcheri/1-470
------------MALF---FVLPGQGRVNQLGGVFINGRPLPNHIRHKIVEMAAAGIRPCVISRQLRVSHGCVSKILCRYQETGSIKPGAIGG
SKPRVATPEVEKKIEDYKRDNPGMFSWEIRDRLLKDGMCDRSTVPSVSSISRILRGGKGDGRRSEDGDSEDERRDGEDGEKKRSHSIDGILGE
KGGCRGEDSDCDSEPDLPLKRKQRRSRTTFTPEQLEELEKAFERTHYPDIYTREELAQRTKLTEARVQVWFSNRRARWRKQAGAQQLAGFNHL
LPMGYPAGATAHASAAYVPLADTSYPITSQVESTTLHRPQPLPPSTVHQSMAADSTSSYGTAGKAYQFTGLSDSFVATAAN----HNTLSGGV
LNILGNPSTCQ-QDYPLASLSTSMDSTQATSSESFTAHAS--THDSYAAH-AQSYCTPP--SYSTTAHPYSMDSYQYGSQYPSQ-GSCTSSYG
STGTSLSSLRQKSLVH--PLGMIPVGSGT-GQAY

>Pax3/7b__A._lucayanum/1-469
------------MALF---FVLPGQGRVNQLGGVFINGRPLPNHIRHKIVEMAAAGIRPCVISRQLRVSHGCVSKXXXXXXXXXXXXXXXXXGG
SKPRVATPEVEKKIEDYKRDNPGMFSWEIRDHLLKDGMCDRSTVPSVSSISRILRGGKGDGRRSDD-DSEDERKDGEDGEKKRSHSIDGILGE
KGGCRGEDSDCDSEPDLPLKRKQRRSRTTFTPEQLDELEKAFERTHYPDIYTREELAQRTKLTEARVQVWFSNRRARWRKQAGAQQLAGFNHL
LPMGYPAGATAHASAAYVPLADTSYPITSQVESTTLHRPQPLPPSTVHQSMAADSTSSYGTAGKAYQFTGLSDSFVATAAN----HNTLSGGV
LNILGNPSTCQ-QEYPLASLSTSMDSTQATSSESFTAHAS--SHDSYAAH-AQSYCTPP--SYSTTAHPYSMDSYQYGSQYPSQ-GXXXXXXX
XXXXXXXXXXXXXXXX--XXXXXXXXXXX-XXXX
```

Supplementary File 4: Python script to map equivalent support values between unadorned .nwk format trees

```
import re
from collections import OrderedDict
from math import log10, floor

try:
    import pyperclip
except ImportError:
    pyper = False
else:
    pyper = True

from comparatordata import *

""" FORMAT TREE FUNCS """

def makespecieslist(speciesstring):
    """ Makes comma-deliniated species string into species list """
    specieslist = speciesstring.split(",")
    specieslist2 = []
    for item in specieslist:
        specieslist2.append(item.replace(" ", ""))
    return specieslist2

def curatespecieslist(specieslist, replacedict):
    """ Removes colons, replaces digits with placeholders, adds inverted commas if missing
"""
    curatedspeciesdict = {}
    for item in specieslist:
        rplace = item.replace(":", "")
        for key in replacedict:
            rplace = rplace.replace(key, replacedict[key])
        rplace = "\'" + rplace + "\'"
        rplace = rplace.replace("\'\'", "\'")
        curatedspeciesdict[item] = rplace
    return curatedspeciesdict

def curatetreespecies(tree, curatedspeciesdict):
    """ Replaces the species names in a tree with the curated species names """
    edited = tree
    for find, replace in curatedspeciesdict.iteritems():
        edited = re.sub(find, replace, edited, 1)
    return edited

def curatetreelist(treelist, curatedspeciesdict):
    """ Replaces the species names in the trees in their list with the curated species names
"""
    curatedtreelist = []
    for tree in treelist:
        edited = curatetreespecies(tree, curatedspeciesdict)
        curatedtreelist.append(edited)
    return curatedtreelist

def curatetrees(specieslistrstr, replacedict, treelist):
    """ Marshals the above funcs into order. """
    specieslist = makespecieslist(specieslistrstr)
    curatedspeciesdict = curatespecieslist(specieslist, replacedict)
    curatedtreelist = curatetreelist(treelist, curatedspeciesdict)
    return curatedtreelist

def uncuratetrees(semifinaltree, replacedict):
    """ Marshals the above funcs to undo curatetrees() and replace the placeholders with
digits"""
    replacedict2 = dict((v, k) for k, v in replacedict.iteritems())
    specieslist = makespecieslist(specieslistrstr)
    curatedspeciesdict = curatespecieslist(specieslist, replacedict)
    uncuratedspeciesdict = dict((v, "\'{}\'".format(k)) for k, v in
curatedspeciesdict.iteritems())
    finaltree = curatetreespecies(semifinaltree, uncuratedspeciesdict)
    return finaltree

""" TOPOLOGY ANALYSIS FUNCS """
```

```
def parenthetic_contents(string):
    """ Generate parenthesized contents in string as pairs (level, contents)."""
    """ Borrowed and modified from Gareth Rees, stackoverflow.com/questions/4284991 """
    """ Takes tree string and returns list of the contents of each node, brackets & commas in
place """
    stack = []
    for i, c in enumerate(string):
        if c == '(':
            stack.append(i)
        elif c == ')' and stack:
            start = stack.pop()
            yield (string[start + 1: i])

def getlistofsupports(string):
    """ Takes tree string and returns list of support values in order - assuming\
    no numbers in seq names. Removes all :###s and then returns all other ###s """
    string2 = re.sub(r':\d+(?:\.\d+)?', '', string)
    return re.findall(r'\d+(?:\.\d+)?', string2)

def splittolists(list1):
    """Takes items in messy list from parenthetic_contents(), cuts out brackets, and\
     makes each item its own sublist of sequence names """
    list12 = []
    for item in list1:
        itemy = str(item)
        itemy2 = itemy.replace("(","").replace(")","")
        itemy3 = itemy2.split(",")
        itemy3.sort()
        list12.append(itemy3)
    return list12

def removeallnumbers(string):
    """ Removes all numbers and numbers preceded with a colon """
    string2 = re.sub(r':?\d+(?:\.\d+)?', '', string)
    return string2

def tuplifyalist(list1):
    """ Takes a list and returns a tuple - necessary to use as dict keys """
    return tuple(tuple(l) for l in list1)

def makeadict(tupley, listy):
    """ Takes a tuple of tuples and a list of lists and makes an ordered dict\
     where each tuple in the tuple is a key for the corresponding sublist """
    dictionary = OrderedDict()
    for n, item in enumerate(tupley):
        if n < len(listy):
            dictionary[item] = listy[n]
    return dictionary

def gofrominputtorightdict(string):
    """ Uses the above functs to go from input string to support value dictionary """
    string2 = removeallnumbers(string)
    listofsupports = getlistofsupports(string)
    list1 = list(parenthetic_contents(string2))
    list2 = splittolists(list1)
    tupleyboy = tuplifyalist(list2)
    dictyboy = makeadict(tupleyboy, listofsupports)
    return dictyboy

def maketreedicts(treelist):
    """ For each tree in the treelist, makes a support value dictionary above using above
funcs """
    dictlist = []
    for tree in treelist:
        treedict = gofrominputtorightdict(tree)
        dictlist.append(treedict)
    return dictlist

def formatsupportvalue(support):
    support2 = float(support)
    if support2 < 0:
        print support2, 'help'
    elif support2 > 0:
        if str(support2)[0:3] != "0.0":
            support2 = round(support2, n-int(floor(log10(float(support2))))-1)
        else:
            support2 = round(support2, n-int(floor(log10(float(support2))))-2)
```

```
    else:
        support2 = "0." + "0"*n
    support2 = str(support2)
    support2 = support2[1:] if support2[0] == "0" else support2
    if len(support) > n+2:
        support2 = support2.ljust(n+1, '0')
    return support2

def makelistofappendedsupports(treedictlist, delinchar, nocladechar, mastertree):
    """ Take list of dictionaries of clades:supports and makes a list of appended supports
"""
    listofappendedsupports = []
    for clade in treedictlist[mastertree]:
        working = []
        for treedict in treedictlist:
            support = treedict[clade] if clade in treedict else nocladechar
            if support != nocladechar:
                support = formatsupportvalue(support)
            working.append(support)
        listofappendedsupports.append(delinchar.join(working))
    return listofappendedsupports

def replacewithappendedsupports(appendedsupports, mastertree):
    """ Takes the master tree and replaces the support values (###:) with the appended
supports in quotations"""
    mastertreeeditlist = []
    edited = mastertree
    for item in appendedsupports:
        edited2 = re.sub(r'\d+(?:\.\d+)?:', '\"{}\"#:'.format(item), edited, 1)
        edited = edited2
        mastertreeeditlist.append(edited2)
    return mastertreeeditlist[-1].replace("#", "")

def formatoutput(finaltree, specieslisstr):
    specieslist = makespecieslist(specieslisstr)
    output = "#NEXUS\nbegin taxa;\n\tdimensions
ntax={0};\n\ttaxlabels".format(len(specieslist))
    for species in specieslist:
        output += "\n\t\'{0}\'".format(species)
    output += "\n;\nend;\n\nbegin trees;\n\ttree mastertree_with_all_supports =
{0}\nend;".format(finaltree)
    return output

def __treecomparator__():
    """ Puts all above function together into core program """
    curatedtreelist = curatetrees(specieslisstr, replacedict, treelist)
    treedictlist = maketreedicts(curatedtreelist)
 #   print treedictlist
    appendedsupports = makelistofappendedsupports(treedictlist, delinchar, nocladechar,
mastertree)
    semifinaltree = replacewithappendedsupports(appendedsupports,
curatedtreelist[mastertree])
    finaltree = uncuratetrees(semifinaltree, replacedict)
    if pyper == True:
        outputty = formatoutput(finaltree, specieslisstr)
        pyperclip.copy(outputty)
        print "Formatted tree copied to clipboard"
    else:
        print finaltree

if __name__ == "__main__":
    __treecomparator__()
```

Supplementary Note: Settings for the phylogenetic analyses

PHYLIP (changes from defaults)

seqboot:

How many replicates?	1000

protdist:

Gamma distribution of rates among positions?	Yes
Analyze multiple data sets?	Yes, 1000 data sets
Coefficient of variation of substitution rate among positions	1.360827635 (= 1 / sqrt(0.54))

neighbor:

Outgroup root?	Yes, at species 1
Randomize input order of species?	Yes (random number seed =192837465)
Analyze multiple data sets?	Yes, 1000 sets

consense:

Trees to be treated as Rooted:	Yes

MEGA-CC (complete contents of the .mao file)

```
    ; Please do not edit this file! If this file is modified, results are unpredictable.
    ; Instead of modifying this file, simply create a new MEGA Analysis Options file by using
the MEGA Prototyper.
    [ MEGAinfo ]
    ver                                   = 7170509-x86_64 Mac OS
    [ DataSettings ]
    datatype                              = snProtein
    MissingBaseSymbol                     = ?
    IdenticalBaseSymbol                   = .
    GapSymbol                             = -
    [ ProcessTypes ]
    ppInfer                               = true
    ppML                                  = true
    [ AnalysisSettings ]
    Analysis                              = Phylogeny Reconstruction
    Statistical Method                    = Maximum Likelihood
    Phylogeny Test                        = ====================
    Test of Phylogeny                     = Bootstrap method
    No. of Bootstrap Replications         = 1000
    Substitution Model                    = ====================
    Substitutions Type                    = Amino acid
    Model/Method                          = JTT with Freqs. (+F) model
    Rates and Patterns                    = ====================
    Rates among Sites                     = Gamma Distributed (G)
    No of Discrete Gamma Categories       = 4
    Data Subset to Use                    = ====================
    Gaps/Missing Data Treatment           = Partial deletion
    Site Coverage Cutoff (%)              = 15
    Tree Inference Options                = ====================
    ML Heuristic Method                   = Nearest-Neighbor-Interchange (NNI)
    Initial Tree for ML                   = Make initial tree automatically (Default -
```

```
NJ/BioNJ)
    Branch Swap Filter                  = None
    System Resource Usage               = ====================
    Number of Threads                   = 4
    Has Time Limit                      = False
    Maximum Execution Time              = -1
```

MrBayes (changes from defaults made in CIPRES interface)

**Maximum Hours to Run:**	168
**My Data Type is:**	protein
**Specify (only) one outgroup:**	6_Bf
**Set the model for among-site rate variation (Rates=)**	gamma
**Set number of rate categories for gamma distribution (Ngammacat=)**	4
**Set the Rate Matrix for Amino Acids (Aamodelpr=)**	Jones
**State Frequencies (Statefreqpr=)**	fixed (empirical)
**Number of Generations (Ngen=)**	500000000
**Stop early if the convergence diagnostic falls below the stop value?**	Yes
**Please enter the stop value**	0.01

The following parameters entered into the command line version should produce an equivalent analysis (untested)

```
prset aamodelpr=fixed(jones) statefreqpr=fixed(empirical)
outgroup 6_Bf
lset rates=gamma ngammacat=4
mcmc ngen=500000000 stoprule=YES stopval=0.01 samplefreq=100 printfreq=100 diagnfreq=1000
```
